# NACs, generalist in plant life

**DOI:** 10.1111/pbi.14161

**Published:** 2023-08-25

**Authors:** Kunjin Han, Ye Zhao, Yuhan Sun, Yun Li

**Affiliations:** ^1^ State Key Laboratory of Tree Genetics and Breeding, Engineering Technology Research Center of Black Locust of National Forestry and Grassland Administration, National Engineering Research Center of Tree Breeding and Ecological Restoration, College of Biological Sciences and Technology Beijing Forestry University Beijing China

**Keywords:** NAC transcription factors, network, plant development, stress

## Abstract

Plant‐specific NAC proteins constitute a major transcription factor family that is well‐known for its roles in plant growth, development, and responses to abiotic and biotic stresses. In recent years, there has been significant progress in understanding the functions of NAC proteins. NAC proteins have a highly conserved DNA‐binding domain; however, their functions are diverse. Previous understanding of the structure of NAC transcription factors can be used as the basis for their functional diversity. NAC transcription factors consist of a target‐binding domain at the N‐terminus and a highly versatile C‐terminal domain that interacts with other proteins. A growing body of research on NAC transcription factors helps us comprehend the intricate signalling network and transcriptional reprogramming facilitated by NAC‐mediated complexes. However, most studies of NAC proteins have been limited to a single function. Here, we discuss the upstream regulators, regulatory components and targets of NAC in the context of their prospective roles in plant improvement strategies via biotechnology intervention, highlighting the importance of the NAC transcription factor family in plants and the need for further research.

## NAC transcription factor family in plants

NAC is one of the largest transcription factor (TF) families in plants (Olsen *et al*., 2005b). NAC proteins are plant‐specific and of special significance to terrestrial plants. The assumed evolutionary order from algae to mosses to monocots and dicots suggests a progression; however, NAC TFs are absent in unicellular green algae, and only sparsely exist in mosses (*Physcomitrella patens*), making the NAC family an interesting research candidate (Bowman *et al*., 2017; Xu *et al*., 2014). More than two decades ago, the first NAC TF was identified in *Petunia* no apical meristem (NAM) (Souer *et al*., 1996). Subsequently, the other two members of NAC, namely the cup‐shaped cotyledons (CUC) protein and the transcription active factor ATAF1/2 in *Arabidopsis*, were discovered (Aida *et al*., 1997; Delessert *et al*., 2005). At present, NAC TFs have been identified in *Arabidopsis*, rice, poplar and other plants, and the number of NAC members contained in each species is different; 117, 151 and 163 NAC genes have been identified in *Arabidopsis*, *Oryza sativa* and *Populus deltoides*, respectively (Hu *et al*., 2010; Nuruzzaman *et al*., 2010; Ooka *et al*., 2003). In 2003, Ooka *et al*. (2003) classified and comprehensively analysed the NAC family members of rice and *Arabidopsis*, and divided the family into two large classes and 18 subgroups (Figure S1). Since the initial comprehensive description of the NAC TF family, further investigations have explored additional functions of NAC TFs in plants. Most NACs are primarily involved in mediating growth and development, while also playing an important role in responding to various stresses (Fang *et al*., 2008; Fujita *et al*., 2004; Kawaura *et al*., 2008). The function and potential mechanism of stress‐related NAC have been extensively studied, but it has been a decade since the last comprehensive summary of NAC response to stress (Puranik *et al*., 2012). Subsequently, research on NAC TFs has developed rapidly. In this review, we discuss the structure and describe how NAC TFs regulate growth and development, including secondary cell wall (SCW) development, seed germination, root development, leaf senescence, flower organ formation and fruit maturity, and mediate plant response to low/high temperature, drought/flooding, high salinity and diseases. Next, we elucidated the molecular mechanism by which NAC TF members regulate plant organ development and related stress responses. Particularly, we propose pivotal NAC factors in developmental and stress responses. This review suggests that a comprehensive understanding of the various regulatory mechanisms of NAC TFs is essential for studying plant TFs.

## NAC, a special plant transcription factor

**Figure 1:**
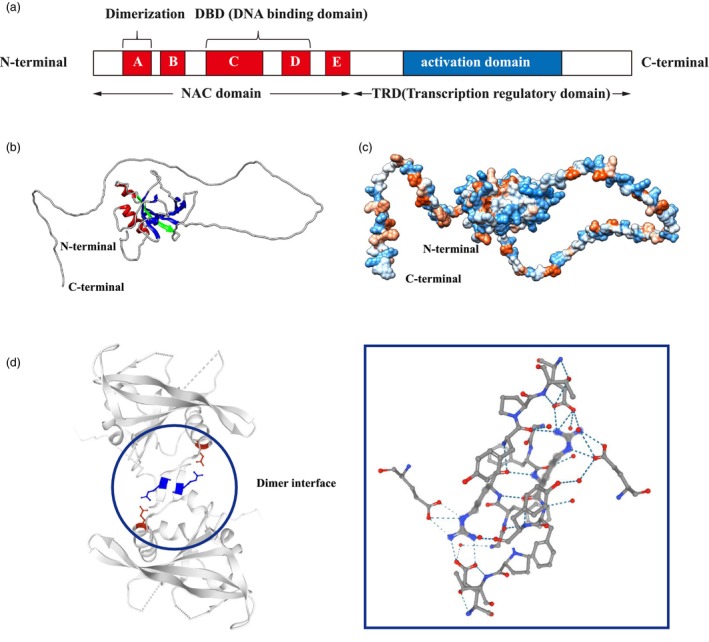
Typical structure of NAC TF. (a) A typical NAC contains a highly conserved NAC domain at the N‐terminal which is divided into five conserved subdomains (A‐E, shown in red). Subdomain A, subdomains B and E, and subdomains C and D are responsible for dimerization, function diversity, and DNA binding, respectively. The C‐terminal region is more diverse and serves as a potential transcriptional regulatory domain (TRD) which has either transcriptional activator or repressor function and sometimes possesses protein binding activity. (b) The domain of the NAC TF ATAF1, secondary structure: the two main helices in red, the main antiparallel b‐sheet in blue and extra strands in green. (c) Hydrophilic and hydrophobic structure of ATAF1 protein. Orange is hydrophobic and blue is hydrophilic. (d) Diagram of the homologous dimer of ANAC019. Figure (b)–(d) made with the programs Chimera (http://www.cgl.ucsf.edu/chimera/).

Generally speaking, all NAC TFs contain a relatively conserved domain and a highly variable transcription regulatory domain (TRD) (Figure 1a). The domain of the classic NAC protein is close to the N‐terminal and consists of approximately 150 amino acid residues. The structure of the NAC domain was determined by X‐ray crystallography, revealing an N‐terminal α‐helix and a short helix on the opposite side. These helices were connected by a twisted antiparallel β‐sheet the typical NAC domain pattern is shown in Figure 1b (Chen *et al*., 2011; Ernst *et al*., 2004; Olsen *et al*., 2005b). The hydrophobicity of the NAC protein ensures structural stability, and hydrophilicity is conducive to the interaction of NAC TFs with other molecules (Figure 1c). The conserved domain can be divided into five subdomains (A to E), with their conservation ranked as A > C > D > B > E (Ernst *et al*., 2004; Olsen *et al*., 2005a). Based on predicted amino acid sequences of the NAC domain, NAC TFs can be categorized into two groups: group I NAC TFs are defined by their conserved B and E subdomains, and group II, which is the opposite (Ooka *et al*., 2003). Subdomain A is involved in the formation of functional dimers, which include homodimers or heterodimers (Figure 1d) (Olsen *et al*., 2004; Puranik *et al*., 2012; Xie *et al*., 2000). Subdomains C and D, which are positively charged contain nuclear location signals and are closely associated with promoter‐specific element binding. The D subunit of several NAC proteins contains a highly hydrophobic negative regulatory domain (NRD). Studies have shown that NARD (NAC Repression Domain) can inhibit the transcriptional activity of TFs such as WRKY and APETALA2 / dehydration‐responsive element (AP2 / DRE) (Hao *et al*., 2010). The conservation of subdomains B and E is relatively weak and determines the functional diversity of the NAC TF (Ernst *et al*., 2004). Typically, the TRD of the NAC protein is located at the C‐terminal and is diverse and intrinsically disordered. There is considerable variation in TRD lengths and sequences among different species, which contributes to the diverse functions of NAC proteins. Furthermore, the TRD region of some NAC proteins has the ability to bind to other proteins (Fang *et al*., 2008; Jensen *et al*., 2010; Tran *et al*., 2010).

A comprehensive phylogenetic analysis was performed on the NAC family genes in *Oryza sativa* (monocot) and *Arabidopsis* (dicot) based on their sub‐domains and bootstrap values (Figure S1). Group I NAC proteins can be further divided into 14 subgroups, including TREN, ONAC022, SENU5, NAP, AtNAC3, ATAF, OsNAC3, NAC2, ANAC011, TIP, OsNAC8, OsNAC7 NAC1 and NAM. Group II NAC proteins were further divided into 4 subgroups (ANAC001, ONAC003, ONAC001 and ANAC063) (Ooka *et al*., 2003). The functions of each of the 18 subgroups corresponded to their classification. For example, considerable research supports the view that NAM and NAC1 subgroups function in development and morphogenesis (Gao *et al*., 2021b; Hendelman *et al*., 2013; Xie *et al*., 2023). ATAF, NAP, AtNAC3 and OsNAC3 showed the function of mediating the resistance to diverse stresses (Delessert *et al*., 2005; Ma *et al*., 2018; Meng *et al*., 2022a). Moreover, certain NAC proteins known as NTLs (NAC with transmembrane motif1‐like, NTM1‐like) possess an α‐helical transmembrane (TM) region, that enables them to anchor to the plasma membrane (Chen *et al*., 2008; Seo *et al*., 2008). Under normal environments, NTLs were not active; however, in stressful environments, they detach from the plasma membrane and enter the nuclear to nucleus to exert their effects (Wang *et al*., 2016). Some atypical NAC proteins exhibit diverse domain numbers and structures, such as the inclusion of two NAC domains, the structural proximity of the NAC domain to C‐terminal, or the TRD to N‐terminal (Puranik *et al*., 2012). Therefore, structural diversity of NAC proteins indicates their involvement in plant growth and development and stress responses.

## Diversity of regulation function of NAC TFs

### Growth and development

#### Development of the SCW

**Figure 2:**
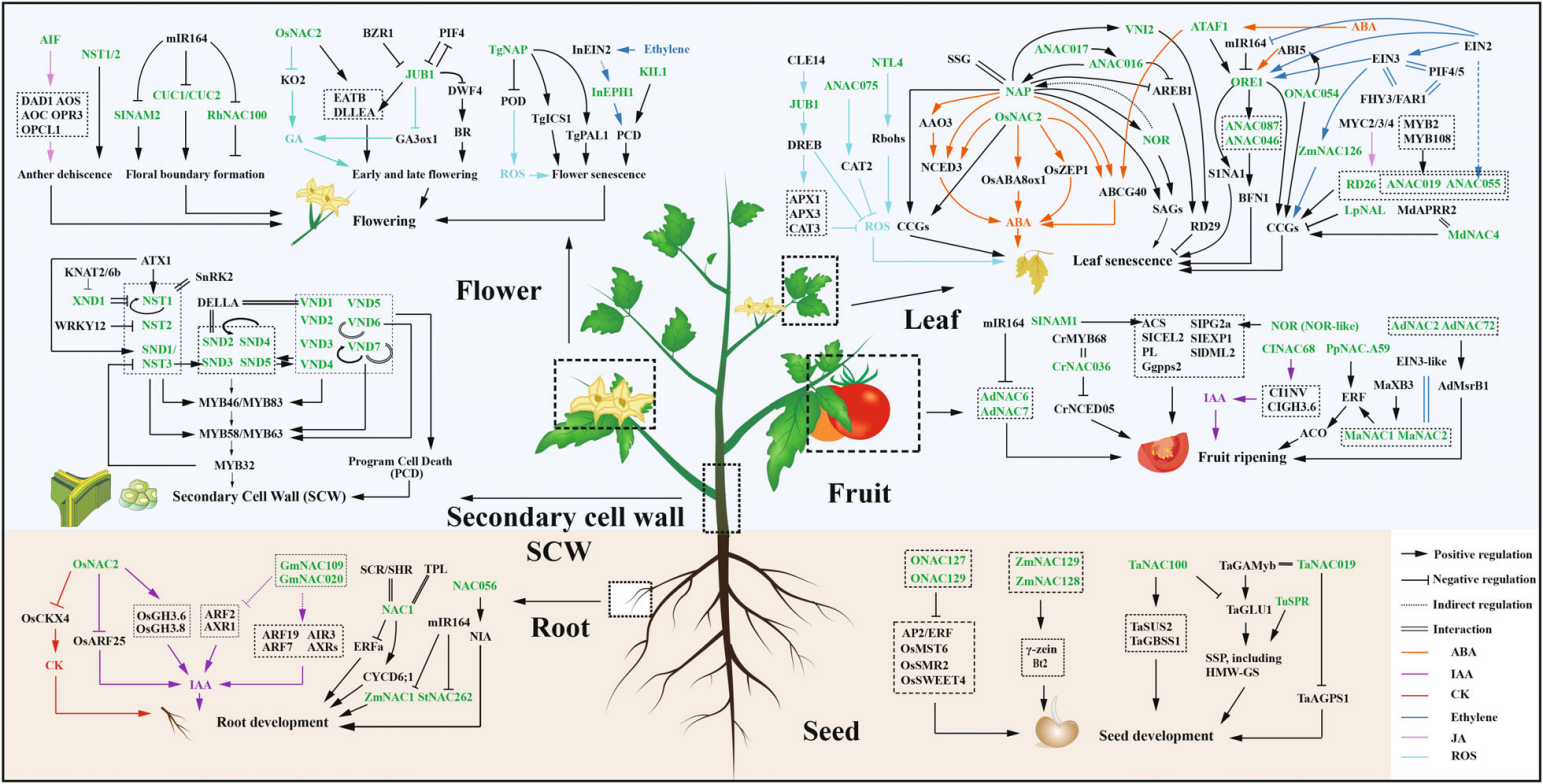
A simplified overview of some regulatory networks that control the development of different plant's parts. The scheme focuses on the transcriptional regulation of plant root, secondary cell wall, leaf, flower, fruit and seed development by NAC TFs. The straight arrow represents activation, the connection of the blunt end represents suppression, parallel lines indicate interactions and dashed lines indicate unknown possible signals. Key to arrow colours: purple, IAA; cyan, ROS; turquoise, GA; orange, ABA; red, CK; blue, ethylene; pink, JA. The green indicates the NAC TFs.

The SCWs are a special type cell type of cell structure, formed through cell extension cessation and subsequent deposition. It serves specific functions in mechanical support and nutrient transport and mainly consists of three components: cellulose, lignin and hemicellulose (Liu *et al*., 2021). NAC TFs act as SCW master switches that play a crucial role in SCW biosynthesis, their crucial roles in this process have been established through an interaction network study in *Arabidopsis* (Taylor‐Teeples *et al*., 2015). Complex interactions between NAC TFs and SCW genes have been observed (Figure 2), with NAC TFs such as VNDs (VND1‐7, VASCULAR NAC DOMAIN), NSTs (NST1‐3, NAC SECONDARY WALL THICKENING PROMOTING FACTOR) and SNDs (SND1‐5, SECONDARY WALL‐ASSOCIATED NAC DOMAIN PROTEIN, SND1 also called NST3) being particularly involved in SCW synthesis (Lee *et al*., 2019; Mitsuda *et al*., 2005, 2007; Tan *et al*., 2018; Zhong *et al*., 2021). VNDs, SNDs and NSTs generally regulate SCW development by binding to a secondary wall NAC binding element (SNBE) (Zhong *et al*., 2010).

In 2002, the first class of NAC family members identified to control SCW development were VNDs, named after their discovery in tracheary elements of *Zinnia elegans* mesophyll cells (Demura *et al*., 2002). In *Arabidopsis*, AtVND6 and AtVND7 modulate SCW formation and programmed cell death (PCD) by participating in xylem formation, which is key to distinguishing VNDs members from other NAC proteins. Moreover, AtVND7 is involved in pectin polysaccharide modification, which has not been observed in NST proteins (Huang *et al*., 2015; Ohashi‐Ito *et al*., 2010; Yamaguchi *et al*., 2011). In cotton, GhVND1 and GhSND2 directly interact with DELLA proteins, which are crucial repressors of gibberellin (GA) signalling, and alter the positive effects of GhNACs on SCW development (Wang *et al*., 2021c).

Plants initiate SCW deposition and lignification by initiating many physiological processes and hormonal signals, including transcription, protein and secondary metabolite synthesis, auxin inhibition, and activation of abscisic acid (ABA) signalling. For example, in *Arabidopsis*, an increase in auxin promoted the expression of *LBD29*, an important TF in lateral growth and callus development processes, which subsequently inhibits the expression of *NST1*, *NST2* and *NST3*, thereby inhibiting SCW growth. In contrast, loss of *LBD29* function resulted in enhanced cell wall biosynthesis in fibres via the upregulated expression of the three main cell wall component biosynthesis genes (Lee *et al*., 2019). In *Arabidopsis*, as a phosphorylated substrate of SnRK2 in the core ABA signalling pathway, AtNST1 positively regulates the transcriptional activation of a series of downstream SCW biosynthetic genes via physical interaction with SnRK2 kinase (Liu *et al*., 2021). In the dicotyledons plant *Cucurbita pepo* L., the mutation of *NST1* resulted in the loss of SCW biosynthesis in the hull‐less seed coat, however, the underlying molecular regulatory mechanism is still not completely understood (Lyu *et al*., 2022). In *Arabidopsis*, the expression of *AtNST1* is controlled by XND1 (XYLEM NAC DOMAIN1), another NAC TF and the interaction of XND1 with NST1 results in the inhibition of *NST1* transactivation, thereby repressing SCW formation (Zhang *et al*., 2020; Zhao *et al*., 2008). In another study, PagKNAT2/6b (KNOTTED‐like homeobox genes) was shown to bind to the *PagXND1* promoter and downregulate its expression to inhibit SCW development (Zhao *et al*., 2020). Additionally, the expression of *AtNST2* is downregulated by AtWRKY12, which inhibits SCW formation (Wang *et al*., 2010).

During the development of SCW biosynthesis, SND1, master transcriptional switch, plays a positive role (Zhong *et al*., 2006). Dominant repression of *AtSND1* in *Arabidopsis* resulted in a drastic reduction in the secondary wall thickening of fibres compared to the wild‐type control. In *Arabidopsis* and *Populus trichocarpa*, SND1 positively regulates SCW development by regulating MYB and other NAC TFs. Studies using an oestrogen‐inducible system revealed that MYB46, SND3 and MYB103 were direct targets of AtSND1. Similar to the contribution of AtSND1 in the regulation of SCW development, PtrSND1 in *Populus* activates the expression of *PtrMYB021* and *PtrMYB074* to regulation SCW deposition (Wang *et al*., 2020a; Zhong *et al*., 2008). Recently, Wang *et al*. (2021a) demonstrated that ATX1 (H3K4‐histone methyltransferase) activates *SND1* and *NST1* expression by increasing H3K4me3 levels and upregulating *SND*1 and *NST1* relative expression during SCW deposition. Similarly, AtSND2/3/4/5 also promotes SCW development, and SND1/2/3/4/5 functions redundantly to regulate SCW thickening (Zhong *et al*., 2021).

NAC TFs often function as the main regulatory factor in regulating SCW development, and the MYB TFs family usually acts as secondary regulatory factors in this regulatory pathway. MYB46 and MYB83 are the two most studied TFs downstream of NAC TFs and they redundantly regulate SCW formation (Zhong *et al*., 2007). They are targeted by AtVND and/or AtNST/SND as second‐layer master switches of SCW biosynthesis (Nakano *et al*., 2015). In addition, NAC TFs regulate other MYB TFs, such as MYB4, MYB7, MYB32, MYB58, MYB63, and MYB103 (Öhman *et al*., 2013; Wang *et al*., 2011; Zhou *et al*., 2009). Notably, MYB TFs not only act as secondary switches regulated by the NAC family but also directly regulate NACs. For example, MYB32 was clarified to inhibit the expression of the NAC TF‐encoding gene *SND1* (Wang *et al*., 2011), forming a negative feedback regulatory pathway.

The above results indicate that the regulatory effects of NAC TFs on SCW development are highly conserved across various plant species. Interestingly, all NAC TFs that have been studied to date positively regulate SCW formation. Among the 12 SCW‐related AtNACs reported to date, 10 belong to the subgroup OsNAC7 (AtNST1‐3, and AtVND1‐7), and 2 belong to ONAC003 (AtSND2/3). Therefore, the expansion of the NAC subgroup OsNAC7 during morphogenesis may be related to SCW development. Identifying additional SCW‐related NACs in diverse species holds promise for future research.

#### Development of seeds

Seed germination marks the beginning of growth and development (Han and Yang, 2015; Smýkal *et al*., 2014). Wheat, corn and rice as the top three global food crops, store essential nutrients such as proteins and starch in their seeds prior to germination (Chen *et al*., 2015b; Evans and Clarke, 2019). In typical monocotyledonous food crops, the endosperm provides sufficient nutrients during seed development (Yang *et al*., 2014). In this section, we discuss the functions of the NACs in terms of their effects on seed development.

In wheat, high‐molecular‐weight glutenin subunits (HMW‐GSs) are the major components of seed storage proteins (SSPs) and largely determine the processing properties of wheat flour. TaNAC100 functions as a central regulator of seed protein and starch synthesis in wheat. Overexpressing *TaNAC100* decreases seed protein levels while increasing starch synthesis. TaNAC100 inhibits the activity of *TaGLU*‐1 (a key seed protein gene, encoding HMW‐GS) and activates the expression of *TaGBSS1* (a key gene regulating seed starch contents) and *TaSUS2* (a key gene of starch synthesis) (Li *et al*., 2021c). The NAC TF TuSPR in wheat binds the cis‐element 5’‐CANNTG‐3′ of the SSP gene promoter and suppresses its expression (Shen *et al*., 2021). The endosperm‐specific TF TaNAC019 from wheat coordinates the accumulation of SSPs and starch by indirectly regulating the expression of *TaSPA* and by directly interacting with the TaGlu‐1 regulators TaGAMyb. Knock‐out of three *TaNAC019* homologues exhibits reduces in transcript levels of all SSP‐type and starch metabolism genes (Gao *et al*., 2021a). Interestingly, when only TaNAC019‐A1 is present, it acts as a negative regulator that directly bind to the ‘ACGCAG’ motif in the promoter regions of *TaAGPS1*‐A1 (ADP‐glucose pyrophosphorylase small subunit 1) and *TaAGPS1*‐B1, repressing their expression, and inhibiting starch synthesis in wheat endosperm (Liu *et al*., 2020a). These results indicate that homologous genes may be functionally diverse. Therefore, it is worthwhile to elucidate the molecular mechanisms by which they synergistically regulate seed germination.

In addition, ZmNAC128 and ZmNAC129 in maize are endosperm‐specific NAC TFs that regulate *Bt2* (*brittle2*, encoding adenosine diphosphate glucose pyrophosphorylase small subunit) and *γ‐zein* (a type of prolamin) transcript, which lower their protein levels, thereby controlling the rate‐limiting step of starch synthesis (Zhang *et al*., 2019). In rice, OsNAC127 and OsNAC129 regulate the seed‐filling process by forming heterodimers. Interestingly, both overexpression and knockdown of these two genes showed incomplete grain filling and shrunken grains. Combined with Chromatin immunoprecipitation (ChIP) revealed that ONAC127 and ONAC129 directly target the monosaccharide transporter gene *OsMST6* and sugar transport gene *OsSWEET4* (Ren *et al*., 2021). In summary, seed germination heavily relies on starch accumulation, proteins and other nutrients, and NAC TFs play an indispensable role in regulating numerous genes associated with seed nutrition.

#### Development of roots

A well‐developed root system is the basis for plant anchoring and absorption of nutrients and water from the soil (Xu *et al*., 2022). The key to continuous root growth is the coordination of cell division and differentiation in meristem. In *Arabidopsis*, *AtNAC1* maintains root meristem and root growth by repressing E2Fa (a major regulator of cell division, DNA repair, and cell differentiation) transcription (Xie and Ding, 2022). Maturation of the basic root tissue depends on the formation of an intermediate cortex that is differentiated from the endodermis A recent study has shown that *AtNAC1* is highly expressed in the basic tissues of primary roots, and the subsequent analysis of the underlying mechanism revealed that AtNAC1 recruits AtTPL (a transcriptional co‐repressor) to the *CYCD6*.1 (cell cycle regulator) promoter to repress transcription. Notably, AtNAC1 was found to interact with AtSCR/SCHR in yeast two‐hybrid system (Y2H), biomolecular fluorescence complementation assay (BiFC), and Co‐Immunoprecipitation (Co‐IP), forming a complex with AtTPL and AtSCR/SCHR. This complex synergistically inhibits the expression of *CYCD6.1*, which influences root tip development via precise timing control (Xie *et al*., 2023). Therefore, AtNACs play an important role in controlling taproot development in *Arabidopsis*.

The roots of dicotyledonous plants are composed of the main and lateral roots, with the lateral root system accounting for most of the root system. In *Arabidopsis*, mutation in *AtNAC056* adversely affects lateral roots' growth, while complementation restores normal phenotypes. Furthermore, AtNAC056 directly binds to the promoter of the nitrate assimilation gene *NIA1*, upregulating its expression and resulting in the promotion of root growth via nitrate signalling (Xu *et al*., 2022). In addition to the nitrate signalling pathway, the auxin signalling pathway is essential for lateral roots. For example, GmNAC20 and GmNAC109 increase Indoleacetic acid (IAA) signalling to promote lateral root growth via the upregulated expression of the IAA‐related genes *ARF7*, *ARF19*, *AIR3*, and *AXRs*, or the downregulated expression of *ARF2* and *AXR1* (Figure 2). Briefly, NAC TFs mediate lateral root growth by increasing IAA production or inhibiting the expression of a series of genes involved in auxin resistance (Hao *et al*., 2011; Yang *et al*., 2019).

In addition to regulating lateral root development in dicotyledonous plants, phytohormone modulates monocotyledonous root development. IAA and cytokinin (CK) act as the primary phytohormones that promote root growth. In rice, the knockout of *OsNAC2* in transgenic plants results in increased root length growth and increased crown roots, suggesting that OsNAC2 is not conducive to root growth. OsNAC2 affects root development by stimulating CK and IAA biosynthesis. Furthermore, OsNAC2 directly up‐regulates the expression of IAA‐inactivity‐related genes (*OsGH3.6* and *OsGH3.8*) and down‐regulated the expression of IAA‐signalling‐related genes (*OsARF25*) and CK oxidation genes (*OsCKX4*), thereby inhibiting the activity and response of IAA and increasing the content of CK (Mao *et al*., 2020a).

During the root growth stage, post‐transcriptional epigenetic modifications play a key role in regulating the number of lateral roots. In maize and potato, miRNAs inhibited the expression of *ZmNAC1* and *StNAC262*, respectively, resulting in a decrease in the number of lateral roots (Figure 2) (Li *et al*., 2012; Zhang *et al*., 2018). Overall, these findings highlight the significant relationship between NAC TFs and root development. However, further investigations are needed to elucidate the underlying mechanisms. Since roots are essential for seed germination and serve as one of the six vital organs, comprehensive studies are required to more thoroughly characterize the mechanism mediating the effects of NACs on root development in various plant species.

#### Leaf senescence

Leaf senescence is a crucial developmental phase where leaves transition from being nutrient‐accumulating organs to nutrient‐exporting organs, facilitating nutrient transfer from ageing leaves to developing organs (Lim *et al*., 2007). Leaf senescence is an essential developmental phase, that is regulated by a series of leaf senescence genes, senescence‐associated genes (SAGs) and chlorophyll catabolic genes (CCGs, including SGR, PAO, NYEs and NYC) (Ma *et al*., 2018). Specifically, phytohormone signalling pathways, including the ABA, ethylene and Jasmonic acid (JA) pathways, play significant roles during leaf senescence.

NAP occupied a pivotal position in the ABA signalling pathway and acts as a positive regulator of ageing (Fan *et al*., 2015; Guo and Gan, 2006; Yang *et al*., 2014). ANAC016 was proven to be a positive regulation factor of the AtNAP upstream, while ANAC016 was also regulated by ANAC017 in *Arabidopsis* (Broda *et al*., 2021; Kim *et al*., 2013). Recently, Xie *et al*. (2022) demonstrated that CCCH zinc finger protein PvSSG interacts with PvNAP1/2 in switchgrass, inhibiting the DNA‐binding ability of PvNAP1/2 and acting as a brake on leaf ageing. In tomato, SlNOR (Non‐ripening) is located in the downstream of SlNAP2 and plays a dual role in leaf senescence and fruit ripening. SlNOR participates in leaf senescence by directly regulating the expression of *SlABCG40*, *SlSAGs and SlCGGs*. Moreover, SlNOR enhances *SlNAP2* expression, suggesting a positively acting feedforward loop involving the two NAC factors (Ma *et al*., 2018, 2019); however, the associated mechanisms remain largely unknown. Thus, it is necessary to study the molecular mechanisms underlying of NOR positive feedback regulation in NAP.

Previous genetic and molecular studies have suggested that, apart from NAP and NOR, multiple NACs mediate leaf senescence via the ABA signalling pathway. In *Arabidopsis*, ATAF1 inhibits and activates the expression of *GLK1* and *ORE1* by directly binding to their promoter, resulting in a shift toward senescence. In addition, ATAF1 directly up‐regulates ABA homeostatic genes, including *NCED3* and *ABCG40* (Garapati *et al*., 2015; Jensen *et al*., 2013). Consistent with the contribution of ATAF1, OsNAC2 induces ABA‐mediated leaf senescence by directly regulating ABA‐biosynthesis gene *NCED* and ABA 8’‐Hydroxylase gene *OsABA8ox1* (Mao *et al*., 2017). In rice, ONAC054 directly regulates CGGs genes and indirectly influences leaf senescence by activating and promoting the expression of *ABA INSENSITIVE5* (*ABI5*) (Sakuraba *et al*., 2020). In summary, ATAF1, OsNAC2, and ONAC054 are key regulators of leaf senescence through the ABA signalling pathway.

NTL4 serves as a representative NAC TF that links the ABA and reactive oxygen species (ROS) signalling pathways. Overexpressing *NTL4* in *Arabidopsis* accelerates leaf senescence, while the NTL4‐deficient mutant *ntl4* exhibit a decrease in ROS levels and delayed senescence. Furthermore, NTL4 is affected by ABA and directly binding to the promoter of the *Atrboh* gene, which encodes ROS biosynthetic enzyme. This binding activates *Atrboh* expression, thereby controlling the ROS pathway, promoting PCD, and accelerating leaf senescence (Lee *et al*., 2012). In contrast, ANAC075 and JUB1 in *Arabidopsis* repress ROS overproduction by directly binding to the promoters of the *CAT2* and dehydration‐responsive element binding protein (DREB) genes, respectively. This binding activates their expression, leading to delayed leaf senescence (Kan *et al*., 2021; Wu *et al*., 2012; Zhang *et al*., 2022b). Moreover, ANAC082, ANAC017 and ANAC090, collectively referred to as the ‘NAC troika’, are extensively involved in the regulatory networks of NAC in ROS and GA signalling pathways, and are closely related to senescence (Kim *et al*., 2018).

The onset of leaf senescence is often influenced by genetics and certain specific stresses. As well‐known stress response factors, NACs play a role in mediating leaf senescence under salt stress. The overexpression of *ORE1* in *Arabidopsis* accelerates salt‐induced leaf senescence (Balazadeh *et al*., 2010). In ethylene‐mediated signalling pathways, *ORE1* is upregulated by EIN2, and EIN3, which are ethylene‐related regulatory factors. EIN2 weakens the effect of miRNA164 on ORE1 transcription (Kim *et al*., 2014; Qiu *et al*., 2015; Sakuraba *et al*., 2014; Xie *et al*., 2021). Additionally, ORE1 interacts with GLK1 and GLK2 to antagonize GLK transcriptional activity, thereby shifting the chloroplast's balance from maintenance to deterioration (Rauf *et al*., 2013a). ORE1 accelerates leaf senescence by directly activating multiple targets, including *SGR1*, *SAG29*, *BFN1* and *SINA1* (Matallana‐Ramirez *et al*., 2013; Ueda *et al*., 2020). Additionally, among the large number of ORE target genes, NAC genes are plentiful, with *ANAC087* being a potential target (Huysmans *et al*., 2018; Kim *et al*., 2014; Vargas‐Hernández *et al*., 2022). ANAC087 and ANAC046 redundantly regulated PCD via BFN1 in root cap development, suggesting that ANAC046 may be a downstream factor of ORE1 in regulating leaf senescence (Huysmans *et al*., 2018).

In the JA‐mediated signalling pathway, three functionally redundant NAC TFs in *Arabidopsis*, *ANAC019*, *ANAC055* and *ANAC072* are activated by the expression of *MYC1/2/3*, a key component of JA signalling, they also mediate JA‐induced chlorophyll degradation by directly regulating CGGs expression (Zhu *et al*., 2015). Additionally, a yeast one‐hybrid assay (Y1H) showed that the promoters of *ANAC019* and *ANAC055* are directly bound by AtMYB2 and AtMYB108 (Hickman *et al*., 2013). Ethylene‐mediated leaf senescence signalling‐related regulation of the NAC TF cascade suggests that EIN2 act as an upstream regulator of ANAC055 and ANAC019, highlighting their potential role in connecting JA and ethylene signalling. *ANAC072*, also known as *RD26*, is a RESPONSIVE TO DEHYDRATION (RD) gene. *RD26* was previously reported to be regulated by the NAC TF VNI2 (ANAC083/VND‐INTERACTING 2) during leaf senescence, and VNI2 itself, is regulated by NAP (Hickman *et al*., 2013; Yang *et al*., 2011). However, the regulation of RD26 by VNI2 requires further investigation.

Moreover, the NAC family members ZmNAC126, BnaNAC60, MdNAC4, and LpNAL also participated in leaf senescence by activating the expression of SAGs and CGGs (Wen *et al*., 2022; Yan *et al*., 2021; Yang *et al*., 2020; Yu *et al*., 2022). Together, NAP, ATAF1, OsNAC2, NTL4, and ORE1 are among the multi‐functional TF regulators that integrate various signalling pathways, including the ABA signalling pathway, ROS, and ethylene signalling pathway, into the leaf senescence program. Therefore, NACs positively and negatively control the leaf senescence process, likely as part of a precise regulatory network that appropriately mediates the termination of leaf life cycles in response to changes in internal and external conditions.

#### Flowering

When the accumulation of energy and nutrients is sufficient, plants enter the reproductive growth stage from the vegetative growth stage, with flowering being a typical feature of this stage in angiosperms. Among the NAC TFs, those involved in the formation of flower boundaries, anther dehiscence, early and late flowering, and flower senescence have been extensively studied (Figure 2).

Flowers are composed of four floral organs: petals, calyx, receptacle, and stamens. The formation of the boundary between floral organs and meristem tissues is important for maintaining the normal development of meristem tissues (Miller *et al*., 2011). Notably, the post‐transcriptional regulation process of NAC TFs is particularly important for the formation of floral boundaries. In tomato model crop, miRNA164‐regulated mutants with inactivated NAM genes produced organ‐fused flowers, whereas the addition of miRNA164‐resistant SlNAM2 to *Slnam* deficient flowers suppressed their fusion phenotype and fully restored the flower border (Hendelman *et al*., 2013). In *Arabidopsis*, CUC1, CUC2 and CUC3 function redundantly in specifying the boundary of the apex, where CUC1 and CUC2 are necessary for initiating of mating primordium and synergistically promote ovule segregation. Loss of CUC1 and CUC2 activity in the *2x35S::MIR164A* line resulted in reduced ovule numbers (Gonçalves *et al*., 2015) In another research, miRNA164 was found to control petal numbers in a non‐redundant manner by regulating the transcript accumulation of the *CUC1* and *CUC2* (Baker *et al*., 2005). These findings demonstrate the overlapping and distinct roles of the homologous TFs CUC1 and CUC2. In contrary to SlNAM2 and CUCs, the abundance of miRNA164 in roses decreases with increasing ethylene production, leading to the accumulation of *RhNAC100* transcripts, which limits cell expansion and reduces petal size (Pei *et al*., 2013). NACs are crucial for regulating floral margin formation, either in a functionally redundant or non‐redundant manner.

The maturation and dehiscence of anthers are important prerequisites for the pollination and fertilization of angiosperms (Ishiguro *et al*., 2001). In the anther dehiscence process of *Arabidopsis*, NST1 and NST2 redundantly regulate SCW thickening of the anther endothelium, which facilitates anther dehiscence and completes anther development. (Mitsuda *et al*., 2007). In the JA‐mediated flowering pathway, *AIF*, an NAC‐like gene, disrupts anther dehiscence by down‐regulating genes involved in JA biosynthesis, such as DAD1, AOS, AOC3, OPR3, OPCL1 (Shih *et al*., 2014).

Plants precisely control flowering time by using different gene regulatory networks to respond to endogenous and exogenous conditions (Ionescu *et al*., 2017). In rice, overexpression of *OsNAC2* results in a late flowering phenotype compared to the wild type, suggesting that OsNAC2 functions as a repressor of flowering time. Further analyses indicated that this late flowering phenotype is caused by OsNAC2 upregulating *OsKO2* and downregulating *OsEATB*, which leads to the inhibition of GA signalling (Chen *et al*., 2015a). In *Arabidopsis*, JUB1 directly represses the expression of the hormone biosynthesis genes *GA3ox1* and *DWF4*, resulting in reduced levels of GAs and BRs. This repression results in late flowering and male sterility. On the other hand, JUB1 activates the DELLA genes *GAI* and *RGL1*, BZR1 and PIF4 act as direct transcriptional repressors upstream of JUB1, establishing a negative feedback loop, and contributing to the late flowering module (Shahnejat‐Bushehri *et al*., 2016).

Ageing in plants is accompanied by PCD (Shibuya *et al*., 2014; Šim Škov *et al*., 2022). In the Japanese morning glory (*Ipomoea nil*), *InEPH1* is regulated by the ethylene pathway factor InEIN2 through an unknown molecular mechanism and regulates downstream PCD genes involved in accelerated flower senescence (Shibuya *et al*., 2014). In maize, KIL1, an ortholog of the *Arabidopsis* NAC TF KIRA1, accelerates the senescence of maize filaments through PCD to terminate flower pollination (Šim Škov *et al*., 2022). In tulips, the overexpression of *TgNAP* accelerates petal senescence, whereas silencing *TgNAP* delays senescence. The specific molecular mechanism behind TgNAP‐induced petal senescence involves the direct activation of SA biosynthesis genes *TgICS1* and *TgPAL1*, and the direct inhibition of *POD12* and *POD17*, which inhibits ROS scavenging (Meng *et al*., 2022b), taken together, TgNAP enhances SA biosynthesis and ROS accumulation to positively regulate petal senescence in tulip.

The NAM subgroup is extensively involved in the regulation of flower development, and its members include SlNAM2, CUC1, CUC2 and OsNAC2. Therefore, other members of this subgroup may also be involved in the regulation of flower development. Elucidating the molecular mechanisms by which other members of this subfamily regulate flowering would be a valuable research endeavour.

#### Fruit ripening

Fruit ripening is a complex, genetically determined process that culminates in dramatic changes in colour, texture, flavour, and aroma (Li *et al*., 2021a). During this process, various biological factors coordinately determine fruit ripening. Understanding how genes control fruit ripening is an outstanding issue in biology. NACs work have been extensively studied for their involvement in fruit ripening, particularly in relation to ethylene signalling (Gao *et al*., 2021b). This study revealed the regulatory roles of NACs in ethylene‐dependent and ‐independent fruit ripening (Figure 2).

Tomato is a commonly utilized model plant in fruit ripening studies because of its well‐characterized genome, stable genetic transformation system, and the availability of CRISPR/Cas9 technology. The onset of fruit ripening is delayed in *slnam1*‐deficient mutants in tomato; conversely, *SlNAM1* overexpressed lines accelerate fruit ripening. Moreover, SINAM1 positively regulates fruit ripening by binding to the promoter of a key ethylene biosynthesis gene, *SlACS*, activating its expression (Gao *et al*., 2021b). NOR can activate *ACS*, *SlGgpps2*, and *SlPL*, which are involved in ethylene biosynthesis, carotenoid accumulation, and fruit softening, respectively. These processes are significantly repressed in CRISPR/Cas9‐edited NOR fruit compared to the wild type, thus delaying fruit ripening (Gao *et al*., 2020). Similarly, NOR1‐like1 accelerates fruit ripening by upregulating the expression of these genes (Gao *et al*., 2018). Moreover, NOR binds directly to the DNA demethylase *SlDML2* promoter and activates its expression, to promote fruit ripening (Gao *et al*., 2022).

Additionally, Shan *et al*. (2012) isolated and characterized six NAC genes, named MaNAC1‐MaNAC6, in banana fruit. These genes exhibit distinct expression trends in the mature pulp and peel. Specifically, in bananas, MaNAC1 and MaNAC2 interact with the ethylene signalling component MaEIL5, an EIN3‐like protein, to participate in fruit ripening (Shan *et al*., 2012). Moreover, MaNAC1 and MaNAC2 negatively regulated *MaXB3* and *MaERF11*, and MaERF11 regulates *ACS1* and *ACO1*. Additionally, *MaNAC2* is inhibited by MaXB3, establishing a negative feedback regulatory pathway (Shan *et al*., 2020). In peach fruit ripening, PpNAC.A59 indirectly promotes ethylene biosynthesis by enhancing *PpERF* expression (Guo *et al*., 2021). NACs may act as upstream of ethylene response factors (ERFs) in ethylene‐mediated signalling regulation.

In kiwifruit, increased ethylene content inhibits miRNA164 activity, which results in the up‐regulation of *AdNAC6* and *AdNAC7* expression, AdNAC6 and AdNAC7 proteins act as transcriptional activators and bind to the promoters of *AdACS1*, *AdACO1*, *AdMAN1* and *AaTPS1*, activating their transcription and promoting fruit ripening (Wang *et al*., 2020b). Moreover, AdNAC2 and AdNAC72 regulate the promoter and transcript of *AdMsrB1*, respectively, with AdMsrB1 being an ethylene‐hypersensitive reductase. This suggests that NAC TFs indirectly regulate the ethylene pathway, leading to increased ethylene content (Fu *et al*., 2021). Therefore, NAC TFs likely modulate fruit ripening via the ethylene pathway.

During ABA‐dependent fruit ripening, CrNAC036 prevents fruit ripening in citrus by synergistically down‐regulating the expression of the ABA pathway gene *CrNCED5* via physical interactions with CrMYB68 (Zhu *et al*., 2020). In the IAA‐mediated pathway, CINAC68 in watermelon positively regulates the accumulation of sugar and IAA by repressing the indole‐3‐acetic acid‐amido synthetase gene *CIGH3*.6 and invertase gene *ClINV*, which controls fruit quality and seed development (Wang *et al*., 2021b). These studies demonstrate the involvement of NAC TFs in fruit ripening through hormone‐transduced signalling pathways, particularly the ethylene pathway, followed by the ABA and IAA signalling pathways. Therefore, future research directions may involve identifying additional NACs related to fruit ripening in hormone signalling pathways and elucidating their molecular mechanisms in regulating fruit ripening.

## Stress response

NAC TF family members have been extensively studied in response to various stresses. The studies on NAC TFs in various stress conditions, including heat, cold, drought, flood, salt, and disease stresses, are summarized below. The regulatory network information is shown in Table 1.

**Table 1:** Molecular and physiological characterization of NAC TFs regulated by stress response.

Species	gene	Subgroup	Expression	Upstream regulators	Target genes	Protein interactions	Features	Title	References
*Arabidopsis thaliana*	*NAC016*	NAC2	Drought		*AREB1*, *NAP*		Negative regulator of drought tolerance	The *Arabidopsis* transcription factor NAC016 promotes drought stress responses by repressing AREB1 transcription through a trifurcate feed‐forward regulatory loop involving NAP.	Sakuraba *et al*. (2015)
	*NAC019*	AtNAC3	Heat, drought, salt, ABA, JA	MYC2, MYBs, ABF3/4, ABI4	*HSFA1b*, *HSFA6b*, *HSFA7a*, *HSFC1*, *ERD1*, *VSP1*, *BSMT1*, *ICS1*	RCF2, ZFHD1	RCF2 dephosphorylates NAC019 in vivo, positive regulator of heat and drought stress	The protein phosphatase RCF2 and its interacting partner NAC019 are critical for heat stress‐responsive gene regulation and thermotolerance in *Arabidopsis*.; A local regulatory network around three NAC transcription factors in stress responses and senescence in *Arabidopsis* leaves.	Guan *et al*. (2014); Hickman *et al*. (2013)
	*NAC055*	AtNAC3	Drought, salt, ABA, JA	MYC2, MYBs, ABF3/4, ABI4	*ERD1*, *VSP1*, *BSMT1*, *ICS1*	ZFHD1	Positive regulator of ABA‐mediated stress signalling, positive regulator of drought tolerance	A local regulatory network around three NAC transcription factors in stress responses and senescence in *Arabidopsis* leaves.	Hickman *et al*. (2013)
	*SHYG*	AtNAC3	Flooding, ET		*ACO5*		Involved in ET‐mediated leaf movement under flooding stress	NAC transcription factor speedy hyponastic growth regulates flooding‐induced leaf movement in *Arabidopsis*	Rauf *et al*. (2013b)
	*JUB1*	ONAC022	Drought	HB13			Positive regulator of drought tolerance	JUNGBRUNNEN1 confers drought tolerance downstream of the HD‐Zip I transcription factor AtHB13	Ebrahimian‐Motlagh *et al*. (2017)
	*RD26/ANAC072*	AtNAC3	Drought, BR		*ARR10*	BIN2	Positive regulator of drought response	GSK3‐like kinase BIN2 phosphorylates RD26 to potentiate drought signalling in *Arabidopsis*.	Jiang *et al*. (2019)
	*ORE1*	NAM	Flooding, disease, ethylene	EIN3	*ACS6*	PevD1	An important regulator of ethylene‐mediated flooding stress, negative regulator of ethylene‐mediated leaf senescence under disease	A stress recovery signalling network for enhanced flooding tolerance in *Arabidopsis thaliana*.; Verticillium dahliae secretory effector PevD1 induces leaf senescence by promoting ORE1‐mediated ethylene biosynthesis.	Yeung *et al*. (2018); Zhang *et al*. (2021)
	*NTL4*	NAC2	Drought		*Rbohs*		Negative regulator of drought tolerance	A NAC transcription factor NTL4 promotes reactive oxygen species production during drought‐induced leaf senescence in *Arabidopsis*	Lee *et al*. (2012)
	*NTL6*	TIP	Salt, disease, ABA			PR1 PR2 PR5	Positive regulator of disease tolerance, negative regulator of ABA‐mediated salt tolerance	Cold activation of a plasma membrane‐tethered NAC transcription factor induces a pathogen resistance response in Arabidopsis; A membrane‐bound NAC transcription factor as an integrator of biotic and abiotic stress signals	Seo *et al*. (2010); Seo and Park (2010)
	*NTL9*	TIP	Disease		*PR1*	CRWN1	Negative regulator of disease tolerance	Lamin‐like proteins negatively regulate plant immunity through NAC WITH TRANSMEMBRANE MOTIF1‐LIKE9 and NONEXPRESSOR OF PR GENES1 in *Arabidopsis thaliana*	Guo *et al*. (2017)
*Betula platyphylla*	*NAC012*	OsNAC7	Salt, osmotic		*P5CS1*, *P5CS2*, *SODs*, *PODs*		Positive regulator of salt tolerance	BpNAC012 positively regulates abiotic stress responses and secondary wall biosynthesis	Hu *et al*. (2019)
*Capsicum annuum*	*NAC1*	NAC2	Cold		*PLD4*		Positive regulator of cold tolerance	Transcription factor CaNAC1 regulates low‐temperature‐induced phospholipid degradation in green bell pepper	Kong *et al*. (2020)
	*NAC2c*	ATAF	Heat, immunity		*HSFA5*	HSP70, NAC029	NAC2c and HSP70 interaction activate thermotolerance NAC2c and NAC029 interaction activate JA‐mediated immunity	Pepper NAC‐type transcription factor NAC2c balances the trade‐off between growth and defence responses	Cai *et al*. (2021)
	*ATAF2*	ATAF	Disease		*PR1*	Hsp26.5	Positive regulator of disease tolerance	Capsicum annum Hsp26.5 promotes defence responses against RNA viruses via ATAF2 but is hijacked as a chaperone for tobamovirus movement protein.	Foong and Paek (2020)
*Glycine max*	*NAC20*	ATAF	Drought, cold, salt		*DREB1A*		Positive regulator of salt and cold tolerance	Soybean NAC transcription factors promote abiotic stress tolerance and lateral root formation in transgenic plants	Hao *et al*. (2011)
	*SIN1*	AtNAC3	Salt, ABA, ROS		*NCED3s*, *RbohBs*		Positive regulator of salt tolerance	A GmSIN1/GmNCED3s/GmRbohBs feed‐forward loop acts as a signal amplifier that regulates root growth in soybean exposed to salt stress	Li *et al*. (2019)
	*NAC181*	NAP	Salt		*NINa*	NSP1a	Positive regulator of salt tolerance and symbiotic nodulation	GmNAC181 promotes symbiotic nodulation and salt tolerance of nodulation by directly regulating GmNINa expression in soybean	Wang *et al*. (2022a)
*Ipomoea batatas*	*NAC3*	NAP	Drought, salt, ABA		*ERA1*, *MREL57*	ANAC011, ANAC072, ANAC083, ANAC100, NAP	ABA‐mediated stress signalling, positive regulator of salt, drought tolerance	The unique sweet potato NAC transcription factor IbNAC3 modulates combined salt and drought stresses.	Meng *et al*. (2022a)
*Lilium longiflorum*	*NAC2*	ATAF	Cold, drought, salt, ABA			DREB1, ZFHD4	Positive regulator of cold, drought, and salt tolerance	A stress‐responsive NAC transcription factor from tiger lily (LlNAC2) interacts with LlDREB1 and LlZHFD4 and enhances various abiotic stress tolerance in Arabidopsis	Yong *et al*. (2019)
	*NAC014*	TIP	Heat		*HSFA3A*, *HSFA3B*, *DREB2B*		Positive regulator of high‐temperature stress	A lily membrane‐associated NAC transcription factor LlNAC014 is involved in thermotolerance via activation of the DREB2‐HSFA3 module	Wu *et al*. (2022a)
*Malus domestic*	*NAC029*	NAP	Cold		*CBF1*, *CBF4*		Negative regulator of cold tolerance in a CBF‐dependent manner	An apple NAC transcription factor negatively regulates cold tolerance via CBF‐dependent pathway	An *et al*. (2018)
*Musa nana Lour*.	*NAC1*	ONAC003	Cold, ethylene, propylene	ICE1		CBF1	Involved in propylene‐mediated cold tolerance	Banana fruit NAC transcription factor MaNAC1 is a direct target of MaICE1 and involved in cold stress through interacting with MaCBF1	Shan *et al*. (2014)
	*NAC5*	NAC1	Disease, SA, MeJA		*PR1‐1*, *PR2*, *PR10c*, *CHIL1*		Involved in GA‐mediated and MeJA‐mediated disease tolerance	Banana fruit NAC transcription factor MaNAC5 cooperates with MaWRKYs to enhance the expression of pathogenesis‐related genes against *Colletotrichum musae*	Shan *et al*. (2016)
	*NAC25*	NAM	Cold		*PLDs*, *DGKs*		Negative regulator of thermotolerance, salt and drought tolerance	NAC‐mediated membrane lipid remodelling negatively regulates fruit cold tolerance	Song *et al*. (2022)
	*NAC28*	NAM	Cold		*PLDs*, *DGKs*, *PLCs*		Negative regulator of thermotolerance, salt and drought tolerance	NAC‐mediated membrane lipid remodelling negatively regulates fruit cold tolerance	Song *et al*. (2022)
*Oryza sativa*	*NTL3*	OsNAC8	Heat		*bZIP74*		Involved in ER protein folding, positive regulator of high‐temperature stress	A membrane‐associated NAC transcription factor OsNTL3 is involved in thermotolerance in rice	Liu *et al*. (2020b)
	*NAC2*	NAM	Drought, salt	SPL10	*AP37*, *COX11*		Positive regulator of drought, salt tolerance	Variations in OsSPL10 confer drought tolerance by directly regulating OsNAC2 expression and ROS production in rice; OsNAC2 positively affects salt‐induced cell death and binds to the OsAP37 and OsCOX11 promoters	Li *et al*. (2022); Mao *et al*. (2018)
	*NAC5*	ATAF	Salt		*NAC6*, *LEA3*, *SNAC1*		Positive regulator of salt tolerance	The abiotic stress‐responsive NAC‐type transcription factor OsNAC5 regulates stress‐inducible genes and stress tolerance in rice The abiotic stress‐responsive NAC‐type transcription factor OsNAC5 regulates stress‐inducible genes and stress tolerance in rice	Takasaki *et al*. (2010)
	*NAC6*	ATAF	Salt		*NAC5*		Positive regulator of salt tolerance		Takasaki *et al*. (2010)
	*NAC14*	‐	Drought		*RAD51A1*		Positive regulator of drought tolerance	Overexpression of OsNAC14 improves drought tolerance in rice.	Shim *et al*. (2018)
	*NAC16*	ONAC003	Drought, ABA, BR	D2	*PUB43*	GSK2, SAPK8	Involved in BR‐regulated rice architecture, ABA‐mediated drought tolerance, negative regulator of drought tolerance	OsNAC016 regulates plant architecture and drought tolerance by interacting with the kinases GSK2 and SAPK8	Wu *et al*. (2022b)
	*ONAC127*	NAC2	Heat		*MST*, *SWEET4*, *MSR2*, *EATB*		High temperature regulates heterodimer formation of ONAC127 and ONAC129 by affecting sugar transport and seed development	A heat stress‐responsive NAC transcription factor heterodimer plays key role in rice grain filling	Ren *et al*. (2021)
	*ONAC129*	AtNAC3							
	*SNAC1*	TIP	Drought, ROS		*SRO1c*, *DST*		Drought sensitivity in deletion mutants, H_2_0_2_ sensitivity in overexpression	The SNAC1‐targeted gene OsSRO1c modulates stomatal closure and oxidative stress tolerance by regulating hydrogen peroxide in rice	You *et al*. (2013)
*Populus alba×P. glandulosa*	*NAC045*	ATAF	Salt, ABA	bHLH104			Positive regulator of salt and ABA tolerance	Functional analysis of PagNAC045 transcription factor that improves salt and ABA tolerance in transgenic tobacco	Zhang *et al*. (2022a)
*Populus tomentosa*	*RD26/ANAC072*	AtNAC3	Drought, BR		*ARR10*		Positive regulator of drought response	Regulation of cytokinin biosynthesis using PtRD26pro ‐IPT module improves drought tolerance through PtARR10‐PtYUC4/5‐mediated reactive oxygen species removal in *Populus*.	Wang *et al*. (2022c)
*Pyrus betulifolia*	*NAC1*	ATAF	Cold, drought			DREB1, DREB2A	Positive regulator of cold, drought tolerance	A novel NAC transcription factor, PbeNAC1, of *Pyrus betulifolia* confers cold and drought tolerance via interacting with PbeDREBs and activating the expression of stress‐responsive genes	Jin *et al*. (2017)
*Poncirus trifoliata*	*NAC72*	AtNAC3	Drought		*ADC*		Negative regulator of drought tolerance	A NAC transcription factor represses putrescine biosynthesis and affects drought tolerance.	Wu *et al*. (2016)
*Rosa chinensis*	*NAC72*	AtNAC3	Drought, ABA	ABF4	*DREB2A*		Positive regulator of drought tolerance, ABA sensitivity in overexpression	Drought‐responsive NAC transcription factor RcNAC72 is recognized by RcABF4, interacts with RcDREB2A to enhance drought tolerance in *Arabidopsis*	Jia *et al*. (2022)
*Solanum lycopersicum*	*NAM3*	NAM	Cold		*ACS1A*, *ACS1B*, *ACO1*, *ACO4*		Involved in ethylene synthesis, positive regulator of cold tolerance	The miR164a‐NAM3 module confers cold tolerance by inducing ethylene production in tomato	Dong *et al*. (2022)
	*JUB1*	ONAC022	Drought		*DREB1*, *DREB2 DELLA*		Positive regulator of drought tolerance	NAC transcription factor JUNGBRUNNEN1 enhances drought tolerance in tomato	Thirumalaikumar *et al*. (2018)
	*VOZ1*	TIP	Drought, ABA		*NGLE FLOWER TRUSS*	OST1, BST	Positive regulator of drought ‐mediated flowering	The tomato OST1‐VOZ1 module regulates drought‐mediated flowering	Chong *et al*. (2022); Selote *et al*. (2018)
	*NAP1*	NAP	Drought, disease, GA, SA, ABA		*GA2ox3*, *PAL3*, *NCED1*		Involved in SA and ABA biosynthesis, involved in GA deactivation, positive regulator of drought disease tolerance	Transcriptomic and genetic approaches reveal an essential role of the NAC transcription factor SlNAP1 in the growth and defence response of tomato	Wang *et al*. (2020c)
*Suaeda liaotungensis*	*NAC10*	NAM	Drought, salt		*P5CS1*, *P5CS2*, *P5CR*		Involved in proline synthesis, positive regulator of drought and salt tolerance	A transcription factor SlNAC10 gene of *Suaeda liaotungensis* regulates proline synthesis and enhances salt and drought tolerance	Du *et al*. (2022)
*Triticum aestivum*	*SNAC8‐6A*	NAP	Drought	ABFs			Positive regulator of drought tolerance	Regulatory changes in TaSNAC8‐6A are associated with drought tolerance in wheat seedlings	Mao *et al*. (2020b)
	*SIP1*	NAC2	Salt		*MDS*	SRO1	Negative regulator of salt tolerance	TaSRO1 plays a dual role in suppressing TaSIP1 to fine‐tune mitochondrial retrograde signalling and enhance salinity stress tolerance	Wang *et al*. (2022b)
*Thellungiella halophile*	*NAC1*	AtNAC3	Heat, drought, salt			HD1	Positive regulator of thermotolerance, salt and drought tolerance	TsHD1 and TsNAC1 cooperatively play roles in plant growth and abiotic stress resistance of *Thellungiella halophile*	Liu *et al*. (2019)
*Vitis vinifera*	*NAC72*	NAP	Disease, SA, ROS		*GLYI‐4*		Involved in SA‐mediated ROS regulation, positive regulator of disease tolerance	Glyoxalase I‐4 functions downstream of NAC72 to modulate downy mildew resistance in grapevine.	Li *et al*. (2021b)
*Zea mays*	*NST3*	OsNAC7	Drought		*CESA5*, *DRP2A*		Positive regulator of drought tolerance	Functions and regulatory framework of ZmNST3 in maize under lodging and drought stress	Ren *et al*. (2020)

*Note*: Indicates no grouping.

### Low‐/high‐temperature stress

**Figure 3:**
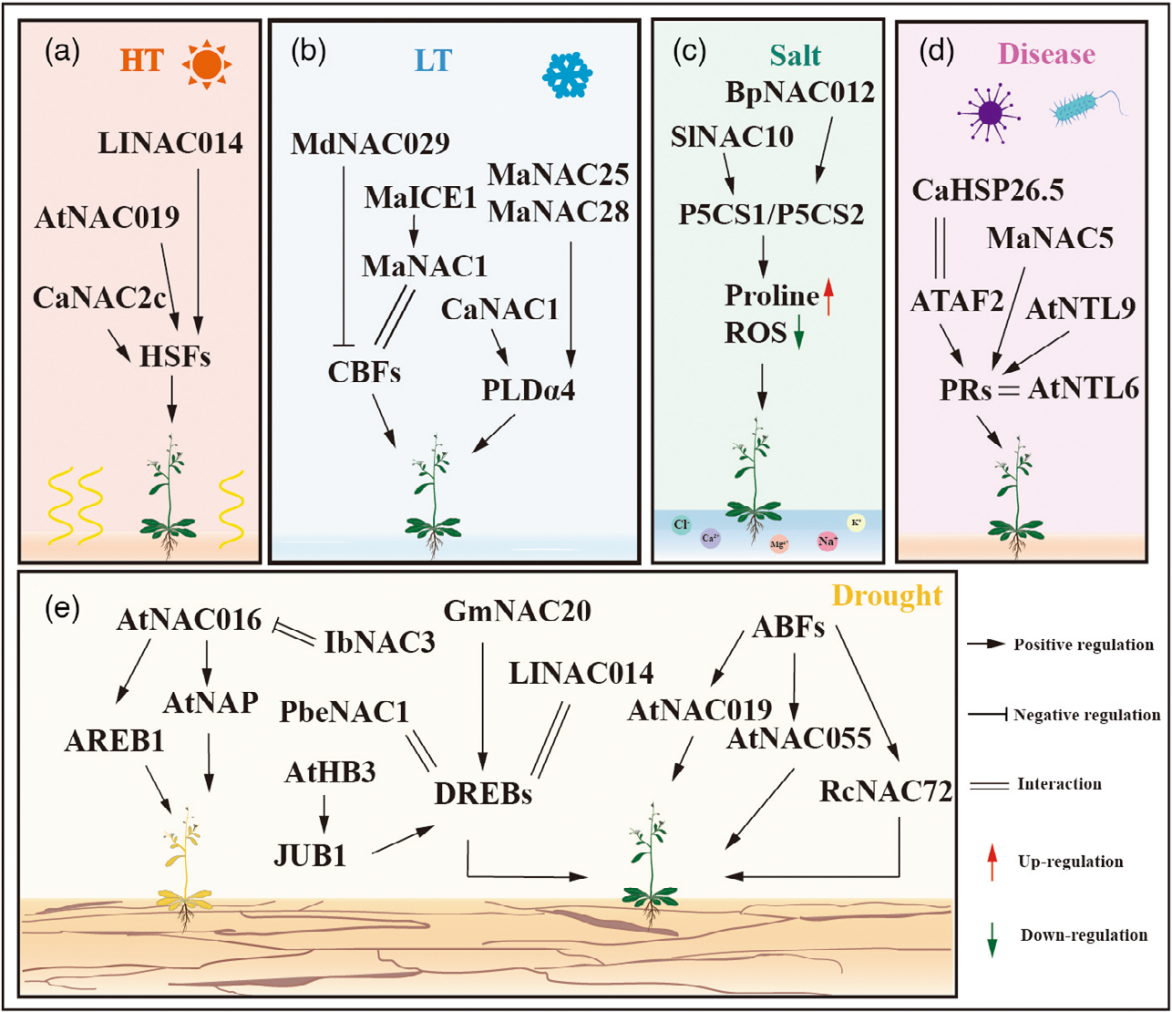
A schematic model of NAC TFs in the regulation of various stresses. (a) NACs regulate heat resistance (HT) in *Arabidopsis thaliana*, *Lilium longiflorum*, *Capsicum annuum*. (b) NACs regulate low temperature (LT) resistance in *Musa nana Lour*, *Malus domestic*, *Capsicum annuum*. (c) NACs regulate salt resistance in *Betula platyphylla*, *Suaeda liaotungensis*. (d) NACs regulate disease resistance in *Arabidopsis thaliana*, *Musa nana Lour*. (e) NACs regulate drought resistance in *Arabidopsis thaliana*, *Glycine max*, *Ipomoea batatas*, *Pyrus betulifolia*, *Lilium longiflorum*, *Pyrus betulifolia*. Arrows represent activation processes, lines ending in bars indicate suppression processes and parallel lines indicate interactions. Red arrows represent increases, green arrows represent decreases.

Low temperatures have significant effects on cellular activity by altering the levels of unsaturated fatty acids and phospholipids. Phospholipid degradation produces under cold stress generates large amounts of phosphatidic acid, which disrupts and alters membrane integrity and fluidity (Shan *et al*., 2014; Song *et al*., 2022). C‐repeat binding factor (CBFs), also known as dehydration‐responsive binding protein 1 (DREB1), is a member of the APETALA2/ethylene responsive factor (AP2/ERF) family and is a key regulator of cold‐responsive genes, especially CBF1, CBF 2 and CBF3. ICE1, a TF inducer of CBF expression, acts as a positive regulator of CBFs and functions upstream in their regulation. NAC TFs function in parallel or in coordination with phospholipid degradation genes or CBF/DREB‐related genes in response to cold stress (Figure 3b) (Yong *et al*., 2019; Zhou *et al*., 2011).

In bananas, MaNAC25 and MaNAC28 negatively regulate cold responses via phospholipid degradation‐related pathways. An analysis of *MaNAC25* and *MaNAC28*‐overexpressing transgenic tomato indicated that MaNAC25 and MaNAC28 bind directly to the promoters of phospholipid degradation genes, including *MaPLDα1/4*, *MaPLDβ1*/2/3, *MaPLDδ1*/2/5, *MaDGK3* and *MaPLC1*/2, forming a positive feedback loop that increases phosphatidic acid accumulation. In contrast, ethylene reduces the activity of MaNAC25 and MaNAC28, thereby inhibiting phospholipid degradation (Song *et al*., 2022) However, overexpression of *SlNAM3* in tomato promotes ethylene production and enhances cold resistance (Dong *et al*., 2022). Therefore, the involvement of NACs in ethylene signalling for regulating cold resistance merits further investigation. In pepper, CaNAC1 directly binds to the *CaPLDα4* promoter and activates *CaPLDα4* transcription, leading to accelerated phospholipid degradation and cell membrane destruction, thereby negatively regulating cold tolerance (Kong *et al*., 2020).

The induction of cold resistance in banana by propylene, an ethylene analogue, reveals the involvement of MaNAC1 in the ICE1‐CBF cold signalling pathway. Y1H and electrophoretic mobility shift assays (EMSA) confirmed that *MaNAC1* functions as a direct target of MalCE1, and it interacts with MaCBF1, a downstream component of MaICE1. These results suggest that MaNAC1 is involved in regulating cold tolerance in bananas (Shan *et al*., 2014). Heterologous overexpression of Lily NAC genes *LlNAC2* in *Arabidopsis* enhances cold resistance by modulation of DREB1/CBF, which binds to the *LlNAC2* (Yong *et al*., 2019). Similarly, soybean NAC genes *GmNAC20* overexpression in *Arabidopsis* enhances cold resistance by activating *DREB/CBF* (Hao *et al*., 2011). Conversely, MdNAC029/MdNAP in apple directly inhibits their expression by binding to the promoters of *MdCBF1* and *MdCBF4* in a CBF‐dependent manner and plays a negative regulatory role in plant cold tolerance (An *et al*., 2018). These results suggest that NACs may act as an intermediate link between ICE1 and CBFs in cold resistance.

High temperature is an important abiotic stress that leads to yield reduction, particularly during the grain‐filling and is easily affected by environmental factors (Bita and Gerats, 2013; Ren *et al*., 2021). As well‐known stress response factors, NACs also help plants escape heat stress through their unique structures or interactions with other factors.

Due to their structural characteristics, certain NAC proteins with transmembrane structures, or the ability to form heterodimers play crucial roles in response to heat stress. For example, LlNAC014 has a typical TM structure at the far end of the C‐terminal and regulates the complex DREB2‐HSFA3 module to enhance thermotolerance. Specifically, LlNAC014 senses high temperatures, transfers to the nucleus, and activates thermotolerance by binding to the promoter cis‐element CTT(N7)AAG of *LlHSFA3A*, *LlHSFA3B*, and *DREB2B* (a dehydration‐responsive element binding protein), resulting in their transcriptional activation (Wu *et al*., 2022a). Heat stress induces the accumulation of misfolded proteins in the endoplasmic reticulum (ER), which initiates the unfolded protein response (UPR) in plants. ER‐related TF OsbZIP74 plays an important role in the UPR in rice. Consistent with the structural features and heat response of LlNAC014 protein structure and heat response way, OsNTL3 in rice directly binds to the promoter of *OsbZIP74* and activates its expression, thus positively responsed to heat stress, in turn, the upregulation of *OsNTL3* under heat stress relies on OsbZIP74 (Liu *et al*., 2020b). Similarly, ZmNAC074 in maize is homologous to OsNTL3 in rice, which has also been shown to positively regulate thermotolerance (Xi *et al*., 2022). However, further investigation is required to determine, whether the molecular mechanisms involved in the heat stress response are those of the same as OsNTL3.

Heterodimers are quaternary protein structures formed by the interaction of two different polypeptide chains linked by disulfide bonds. They represent a special form of protein interaction that plays a unique role in stress response regulation (Ren *et al*., 2021; Yao *et al*., 2022). During rice reproduction, ONAC127 and ONAC129 regulate the heat stress response by forming heterodimers and targeting the calmodulin‐like protein gene *OsMSR2* and the AP2/ERF factor gene *OsEATB*. They coordinate grain filling regulation under high temperature (Ren *et al*., 2021). Moreover, the dimerization between the subdomain of NAC and other TFs plays an important role in the heat stress response. For example, TsHD1 cloned from the halophyte *Thellungiella halophila*, is a homeodomain (HD) TF gene that can improve heat tolerance by forming heterodimers through the interaction between the TsHD1 zinc finger domain and the A subdomain of TsNAC1 (Liu *et al*., 2019).

Heat shock TFs (HSFs) and heat shock proteins (HSPs), which act as molecular chaperones for HSFs, are key TFs that respond to heat stress, NACs are likely to function as upstream regulators of HSFs (Figure 3a). In *Arabidopsis*, AtNAC019 interacts with regulators of C‐ repeat binding factor (RCFs) and undergoes dephosphorylation by RCF2, and then binds to the promoter of *HSFA1b*, *HSFA6b*, *HSFA7*, and *HSFC1* to positively regulate heat tolerance (Guan *et al*., 2014). Thus, AtNAC019 is a critical mediator linking RCFs and HSFs during heat stress response in *Arabidopsis*. In pepper, the interaction between CaNAC2c and CaHSP70 in the nucleus protects CaNAC2c from degradation, thus allowing CaNAC2c to bind to the target factor *CaHSFA5* and activates its transcription. Additionally, this interaction blocks H_2_O_2_ accumulation, thereby enhancing thermotolerance (Cai *et al*., 2021).

When NAC TFs respond to low‐ and high‐temperature stresses, their cofactors are different. For example, NAC TFs are mostly related to HSF TFs when regulating heat stress and are mostly related to CBFs when regulating cold stress. Notably, there exist subtle links between NAC TFs and CBFs under cold and heat stresses. Investigating the synergy and regulation between NACs and CBFs during these stresses presents an interesting research challenge. Furthermore, based on the subgroup, LlNAC2 and GmNAC20 belong to the ATAF subgroup, MdNAC029 belongs to the NAP subgroup, AtNAC019, ONAC129 and TsNAC1 belong to the AtNAC3 subgroup. These subgroups are adjacent to each other in terms of grouping (An *et al*., 2018; Guan *et al*., 2014; Hao *et al*., 2011; Liu *et al*., 2019; Ren *et al*., 2021; Yong *et al*., 2019). It is speculated that these three subfamilies of the NAC family are closely related to low‐ and high‐temperature stresses. Briefly, precise modulation of the aforementioned NAC proteins contributes to enhancing cold or heat resistance in plants.

### Drought and flooding stress

Drought can have a devastating effect on plant growth and crop yield, which is usually caused by the accumulation of cell‐damaging ROS and the inhibition of photosynthesis (Suzuki *et al*., 2014; Thirumalaikumar *et al*., 2018). ABA‐mediated signalling pathways have been found to be closely associated with drought (Jia *et al*., 2022; Wang *et al*., 2020c). Members of the NAC family reportedly regulate drought tolerance through various pathways and synergistically regulate many factors, such as ABA‐responsive element binding protein (AREB), ABA‐responsive element binding factor (ARF), and DREB (Figure 3e). Moreover, the epigenetic modification of NAC protein also plays an important role under drought stress (Selote *et al*., 2018; Sosa‐Valencia *et al*., 2017).

**Figure 4:**
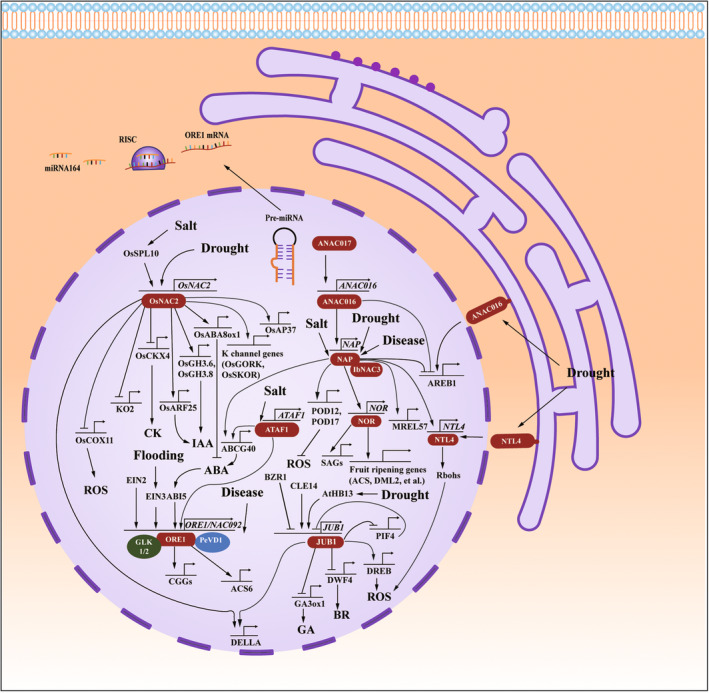
Schematic representation of possible roles for NAC TFs in integrating abiotic and biotic stress responses. Schematic representation of the possible roles of key NACs in integrating plant development and stress responses. OsNAC2 is involved in plant lateral root and flower organ development, as well as leaf senescence and regulation of various abiotic stresses. NAP is involved in flower organ development, leaf senescence and abiotic stress caused by cold damage. ATAF1 is involved in lateral root development, leaf senescence and disease stress regulation. Expression of *ORE1* is negatively regulated by miRNA164 at the post‐transcriptional level, ORE1 is involved in leaf senescence, flooding response, and disease stress regulation, JUB1 is involved in leaf senescence, flower organ development and drought stress regulation.

The ABA‐dependent pathway plays a major role in the drought stress response, and the ABA response element ABRE is involved in ABA‐regulated gene expression (Yoshida *et al*., 2014). In *Arabidopsis*, under drought stress, the NAC TFs AtNAC016 and AtNAP along with the ABA TF AREB1, enhance the plant's drought response to drought stress through a tridentate feedforward pathway. Specifically, AtNAC016 directly binds to the promoter of *AREB1* and inhibits its transcription, AtNAC016 also directly targets *AtNAP*, which similarly regulates *AtAREB1*. Moreover, mutants of *AtNAC016* and *AtNAP* exhibit increased drought tolerance, while overexpression lines showed opposite results (Sakuraba *et al*., 2015). This suggests that NAC016 and NAP reduce plant drought tolerance by negatively regulating the ABA signalling pathway. Interestingly, NAP functions differently in regulating stress response between *Arabidopsis* and tomato. In tomato, *SlNAP1* overexpression dramatically improved drought tolerance. EMSA and chromatin immunoprecipitation‐quantitative PCR (ChIP‐qPCR) assays indicated that SlNAP directly activated the transcription of the GA inactivation‐related gene *SlGA2ox3* and the SA synthesis‐related gene, moreover, SlNAP positively enhances drought tolerance in tomato by activating the ABA synthesis‐related gene *SlNCED1* (Figure 4; Wang *et al*., 2020c). Their versatile functions may have evolved to ensure plant survival under diverse stress conditions. Taken together, these results indicate that NAP plays different roles in controlling drought resistance across different plants.

ABFs and AREBs belong to the bZIP class of TFs and play equally important roles in the drought response as key factors in the ABA signalling pathway. In roses, *RcNAC72* expression is induced by multiple stresses including drought. Overexpression of *RcNAC72* in *Arabidopsis* enhances drought stress by interacting with RcDREB1A (Jia *et al*., 2022). The drought‐tolerance‐related gene *TaSNAC8‐*6A was isolated from the wheat genome by candidate gene association analysis, revealing the ABF binding sites in its promoter region, ABF enhances the drought‐induced expression of *TaSNAC8*‐6A in drought‐tolerant genotypes (Mao *et al*., 2020b).

Genetic and molecular studies suggested that NAC TFs mediate drought tolerance via distinct molecular mechanisms. *AtJUB1* overexpression in *Arabidopsis* significantly increases drought tolerance, and its promoter contains a binding site for AtHB13 (HD‐Zip I protein), indicating their interaction. (Ebrahimian‐Motlagh *et al*., 2017). Similarly, SlJUB1 in tomatoes positively regulates plant drought tolerance by directly binding to the promoter of *SlDREB1*, *SlDREB2* and *SlDELLA* activating their expression (Thirumalaikumar *et al*., 2018). Thus, the HB‐JUB1‐DREB/DELLA pathway plays a key role in drought stress tolerance.

Drought tolerance is also affected by ROS levels. Rice OsNAC2 positively regulates drought resistance by inhibiting ROS accumulation. It indirectly regulates PCD by activating *OsAP37* to activate caspase activity and by inhibiting *OsCOX11* to inhibit ROS accumulation, thereby improving plant drought tolerance. The promoter of *OsNAC2* is directly bound by the OsSPL10 TF leading to upregulated expression of *OsNAC2* (Li *et al*., 2022). In contrast, rice SNAC1 and *Poncirus trifoliata* PtrNAC72, negatively regulate plant drought tolerance by targeting *OsSRO1c* (a stress‐related plant‐specific protein) and *PtACD* (a putrescine biosynthesis factor) leading to increased ROS accumulation (Wu *et al*., 2016; You *et al*., 2013). Thus, NAC TFs suppress and promote drought tolerance in plants by positively and negatively regulating ROS accumulation.

Epigenetic modifications include post‐transcriptional and post‐translational modifications. Post‐transcriptional regulation is generally affected by microRNA, which cleave mRNA or inhibits mRNA translation. In *Phaseolus* vulgaris, the NAC TF *Phvul*.010 g120700 is targeted by miRNA1514a. Overexpression of miRNA1514a results in reduced accumulation of *Phvul.010 g120700* and an increase in NAC‐derived phasiRNA, ultimately promoting drought resistance in plants (Sosa‐Valencia *et al*., 2017). *OMTN1*‐*OMTN6* are six conserved NAC genes in rice, and miRNA164 targets and activates four of them, namely *OMTN2*, *OMTN3*, *OMTN4* and *OMTN6*, to negatively regulate drought resistance genes (Fang *et al*., 2014). Post‐translational regulation, including ubiquitination and phosphorylation also affect the regulation of NAC TFs during drought stress response. Overexpression of *OsNAC016* attenuates drought tolerance and reduces ABA sensitivity, whereas mutations have the opposite effect. SAPK8, which is closely related to the ABA pathway, phosphorylates OsNAC016 and reduces its stability, and then OsPUB43 ubiquitin proteasome promotes the degradation of OsNAC016, leading to the improved drought tolerance (Wu *et al*., 2022b). Similarly, phosphorylation and ubiquitination are also involved in the modification of the NAC TFs RD26 in *Arabidopsis* and SlVOZ1/2 in tomato, regulating drought tolerance in plants (Chong *et al*., 2022; Jiang *et al*., 2019; Selote *et al*., 2018).

Flooding has detrimental effects on biodiversity, natural species distribution, and global food production (Normile, 2008; Silvertown *et al*., 1999). Hydrologically defined niches are the basis for species richness in plant communities. Flooding primarily causes hypoxic stress, and plants respond differently to hypoxic stress (Zhou *et al*., 2020). In rosette plant *Arabidopsis*, overexpression of the NAC TF *SHYG* triggers, a rapid upward leaf movement, in response to stress by directly targeting the key ethylene biosynthesis enzyme gene *ACO5* (Rauf *et al*., 2013b). Under flooding stress, increased ethylene content induces the ethylene response factor EIN3, which, in turn, increases the expression of *ORE1*, and accelerates leaf senescence (Yeung *et al*., 2018). ANAC102 is a specific factor related to low‐oxygen stress during seed germination under flooding conditions. Repressing the expression of *ANAC102* decreases the germination rate, while, *ANAC102* overexpression has no significant effect on germination (Christianson *et al*., 2009). Further research is warranted to elucidate the precise molecular regulatory mechanisms involved.

The ABA and ethylene signalling pathways are the main pathways through which NAC proteins respond to drought and flooding stress, respectively. The NAP (AtNAP), AtNAC3 (RD26 and SHYG) and ATAF (ANAC102) subgroups are also involved in drought and flood stress responses (Christianson *et al*., 2009; Jiang *et al*., 2019; Rauf *et al*., 2013b; Sakuraba *et al*., 2015). Additionally, the NAC2 subgroup members AtNAC016, ONAC022 subgroup member JUB1, and NAM subgroup member ORE1 are also involved in drought and flooding stress responses (Ebrahimian‐Motlagh *et al*., 2017; Sakuraba *et al*., 2015; Yeung *et al*., 2018). However, research on the molecular mechanisms of NAC involvement in flooding stress remains limited, highlighting a unique direction for future research.

### Salt stress

Globally, more than 6% of the land area is increasingly being affected by salt accumulation, posing harm plant growth, crop production and ecosystem balance. (Ismail and Horie, 2017; Munns and Tester, 2008; Van Zelm *et al*., 2020). Salt stress has been widely studied in a variety of plants, such as *Arabidopsis*, wheat, rice, soybean, and *Populus* (Table 1), which have evolved multiple mechanisms to resist salt stress, including osmoregulation, ROS scavenging, ion homeostasis and regionalization, and phytohormone regulation (Van Zelm *et al*., 2020).

Proline is an osmoregulatory substance that plays an important role in protecting the photosynthetic system and enhancing the antioxidant system of plants (Van Zelm *et al*., 2020). For example, in the *halophyte Suaeda liaotungensis* K., *SlNAC10* expression is induced by salt, drought, cold, and ABA. Overexpression of *SlNAC10* in *Arabidopsis* enhances salt stress tolerance and subsequent exploration showed that SlNAC10 improves salt tolerance by binding to the promoter of the proline synthesis‐related enzyme genes *AtP5CS1*, *AtP5CS2*, *AtP5CR* and regulating their downstream gene transcription (Du *et al*., 2022). Furthermore, saline conditions induced the expression of *BpNAC012* in *Betula platyphylla* and *AtJUB1* in *Arabidopsis*. Both *BpNAC012* and *AtJUB1* encode positive regulators of salt stress tolerance and are closely associated with osmoregulation (Alshareef *et al*., 2019; Hu *et al*., 2019).

In rice, OsNAC2 plays a negative role in the regulation of salt tolerance regulation. Overexpression of *OsNAC2* causes nuclear DNA fragmentation, alters caspase‐like activity, and accelerates salt‐induced cell death. A series of factors that are not conducive to cell survival include OsNAC2 targets genes encoding a ROS scavenger (*OsCOX11*), a caspase‐like protease (*OsAP37*), and activated K^+^‐efflux channel genes *OsGORK* and *OsSKOR*, then resulting in ROS accumulation and ion imbalance (Mao *et al*., 2018). In summary, OsNAC2 negatively regulates salt stress tolerance via ROS and ion‐related pathway.

Under salt stress conditions, overexpressing *ATAF1* in *Arabidopsis* exhibits increased sensitivity to oxidative and salt stress, indicating the involvement of ATAF1‐mediated salt response signalling pathway in ROS during abiotic stress (Wu *et al*., 2009). Interestingly, the functions of the homologous genes in different plants are reversed under salt stress. *GmNAC109* in soybean is homologous to *ATAF1* in *Arabidopsis*, and salt stress can significantly induce the expression of soybean *GmNAC109*, whereas overexpression of *GmNAC109* in *Arabidopsis* enhances salt tolerance compared to wild‐type (Yang *et al*., 2019). The opposite behaviour of ATAF1 under salt may be attributed to salt‐induced changes in other unknown factors, which require further investigation. In a separate study, GmSIN1 (salt‐induced NAC1) enhances soybean salt tolerance via a combined modulation pathway of ROS and ABA. An analysis of *GmSIN1*‐overexpressing transgenic soybean indicated that GmSIN1 directly binds to the promoters of *GmRbohBs* (*Respiratory burst oxidase homologue B genes* associated with ROS generation) and *GmNCED3s* (associated with ABA synthesis), upregulating their expression and resulting in the rapid accumulation of ABA and ROS. *GmSIN1*, *GmNCED3s* and *GmRbohBs* constitute a positive feedforward system that, effectively amplifies the initial salt stress signal, enhancing soybean salt stress tolerance (Li *et al*., 2019).

Salt stress is closely associated with the ABA pathway (Zhu, 2002). Meng *et al*. (2022a) demonstrated that ectopic expression of *IbNAC3* (a sweet potato NAC TF) in *Arabidopsis* confers tolerance to complex stresses, including salt stress, by integrating multiple pathways including ABA. In the ABA regulatory pathway, IbNAC3 directly promotes the transcription of *ERA1* a key negative regulator of ABA signalling, resulting in reduced ABA sensitivity, and enhanced salt resistance in plants. Moreover, overexpressing *ONAC022* in rice confers extreme salt tolerance, accompanied by upregulated expression of ABA biosynthesis genes, *OsNCEDs*, and *OsPSY* (Hong *et al*., 2016). DlNAC1, a member of the ONAC022 subgroup in *Dendranthema lavandulifolium*, regulates salt tolerance in a similar way (Yang *et al*., 2016).

In addition to the aforementioned key regulatory mechanisms, several other genes are closely related to NAC TFs. For example, mitochondrial retrograde signalling has long been considered critical for stress perception in eukaryotes. Under salt stress, the NAC TF TaSIP1 in bread wheat translocates from the ER to the nucleus and activates several other mitochondrial dysfunction stimulation (MDS) genes. Overexpressing *TaSIP1* in wheat compromises plant stress tolerance to salt stress. In contrast, the main agronomic factor TaSRO1 interacted with TaSIP in the cytoplasm, causing more *TaSIP1* to be blocked in the ER membrane and nucleus, thereby attenuating the trans‐activating activity of TaSIP1 and thus reducing MDS activation (Wang *et al*., 2022b), thus suggesting that the TaSRO1‐TaSIP1 module balances growth and stress responses by fine‐tuning the level of mitochondrial retrograde signalling. In rice, OsNAC5 enhances salt stress tolerance by interacting with OsNAC6, which synergistically upregulates the ‘late embryogenesis abundant’ gene *OsLEA3* (Takasaki *et al*., 2010).

Collectively, the results of analyses of model plants indicate that the regulatory effects of NACs on salt stress response are highly conserved, although their regulatory pathways in response to salt stress signalling may vary. Among the 13 NAC genes related to the salt stress response, 6 belong to NAP (1), AtNAC3 (1), and ATAF (4) subgroups. The remaining seven genes are found in the NAM (2), OsNAC7 (1), ONAC022 (3) and NAC2 (1) subgroups (Table 1). Strikingly, all these subgroups belonged to group I, suggesting that group I is closely associated with salt stress. Moreover, the mechanisms of NACs which regulate salt stress primarily involve osmoregulation, ROS scavenging and phytohormone regulation, whereas ion homeostasis and compartmentalization are less well‐studied, phytohormone regulation is predominantly focused on ABA signalling. Exploring the mechanisms underlying ion homeostasis and compartmentalization, and other phytohormone regulations presents an interesting challenge for future studies.

### Disease stress

Plants face constant challenges from various organisms, such as viruses, bacteria, fungi, oomycetes, herbivores, and parasitic plants, which can influence the occurrence of diseases. Consequently, plants have evolved strong immune defence mechanisms (Ngou *et al*., 2022). Understanding the control of disease defence mechanisms by NAC genes is crucial. NAC TFs play extensive roles in disease regulation, involving diverse and complex signalling pathways, such as salicylic acid (SA), methyl jasmonate (MeJA), ABA, and ethylene signalling (Table 1).

In plant disease resistance, SA is considered as the main defence hormone against biotrophic and semi‐biotrophic pathogenic infections. For example, *SlNAP1*‐overexpressing tomato lines showed significantly enhanced defence against two widespread bacterial diseases, namely leaf speck disease and root‐borne bacterial wilt disease, with disease resistance being related to the SA pathway. Furthermore, SlANP1 binds directly to the *SlPAL3* promoter by recognizing the NAC core binding site CACG, thereby activating *SlPAL3* transcription to promote SA biosynthesis (Wang *et al*., 2020c). In grapes, overexpression of *VvNAC72* enhances downy mildew tolerance via SA‐mediated ROS pathway. The specific mechanism involves VvNAC72 inhibiting the transcription of glyoxalase *VaGLYI*‐4, disrupting cytotoxic methylglyoxal production, and significantly increasing the expression of SA‐related genes, thereby improving disease resistance (Li *et al*., 2021b).

Membrane‐associated NAC TFs are also closely linked to disease defence. AtNTL9, which is associated with SA synthesis, forms protein complexes with AtCRWN1 and AtSNI1 in *Arabidopsis*. Together, they interact with and repress the transcription of the defence gene *AtPR1*, thereby negatively regulating immunity (Guo *et al*., 2017). Notably, AtNTL6 mediates cold‐induced expression of PR genes mediates cold‐induced expression of PRs genes (*AtPR1*, *AtPR2*, and *AtPR5*) independently of SA (Seo *et al*., 2010). Therefore, membrane‐associated NACs regulate plant disease resistance in an SA‐dependent or independent manner.

MeJA plays an important role in response to biotic stresses. In banana, SA and MeJA treatments significantly enhance *MaNAC5* expression. Moreover, MaNAC5, MaWRKY1 and MaWRKY2 act as transcriptional activators, individually or together, to activate the transcriptional activities of *MaPR1‐1*, *MaPR2*, *MaPR10c* and *MaCHIL1* (*chitinase‐like*) genes, inducing resistance against *Colletotrichum musae* (Shan *et al*., 2016), This highlights the crucial role of MaNAC5 as a connecting hub of MeJA and SA pathways in disease resistance and stress responses. Further research on its related mechanisms would be highly valuable.

ABA and ethylene signal pathway also participate in disease regulatory response. Premature leaf senescence in Arabidopsis is caused by the soil‐borne vascular fungus *Verticillium dahlia*, while virus‐induced gene silencing of *GhORE1* delays *V. dahlia*‐induced leaf senescence in cotton. Investigation into ORE1 regulation of pathogen‐induced senescence found that GhORE1 stability is influenced by a protein exciton (PevD1), which inhibits GhORE1 ubiquitination by the cyclic ubiquitin E3 ligase NLA, subsequently inhibiting the expression of *ACS6* (an ethylene biosynthetic gene) downstream of ORE1 and reducing ethylene synthesis. Additionally, ORE1 has been associated with ABA during leaf senescence in a previous study (Garapati *et al*., 2015). Therefore, it is reasonable to postulate that ABA induces leaf senescence via the ORE1‐ACS6 module. Additionally, in *Arabidopsis*, histological analysis of the *ATAF1* mutant inoculated with *Blumeria graminis* f. sp. *hordei* (*Bgh*) showed that ataf1 significantly induced the expression of the ABA biosynthetic gene *AAO3*, which regulates plant resistance to the pathogen through the ABA pathway (Jensen *et al*., 2008). Thus, NACs positively and negatively regulate disease defence, likely as part of a precise regulatory network that appropriately mediates defence in response to changes in biological stress.

### Development and stress response NAC hub

Plants’ life encompasses various stages, starting from seedling emergence and progressing through growth, ageing and eventual death. This process involves intricate gene activities that orchestrate cell growth, and tissue development, and the maturation of the development of vegetative organs and reproductive organs. Over time, different plant organs undergo ageing until their demise and finally the ageing of various plant organs of plants until death (Du and Jiao, 2020; Thomas, 2013). Throughout their growth journey, plants inevitably experience adverse environmental conditions, including abiotic and biotic stresses, and have evolved unique mechanisms to perceive and respond to these stresses (Zhu, 2016). NAC TFs play a pivotal role in regulating and coordinating other factors to mediate both plant development and stress responses. A notable example of this interplay is observed in a regulatory network involving OsNAC2, NAP, ATAF1, ORE1, and JUB1 which integrates various stress signals within the context of plant developmental programs (Figure 4).

OsNAC2 interacts with a wide range of signals to participate in plant development in response to stress. OsNAC2 inhibits root development through IAA and CK signalling (Mao *et al*., 2020a). Moreover, OsNAC2 positively regulates leaf senescence via the ABA signalling pathway (Mao *et al*., 2017), and delays flowering via the GA pathway (Chen *et al*., 2015a). In terms of stress response, OsNAC2 is closely related to the ROS pathway and participates in salt stress responses through ion‐related pathways (Mao *et al*., 2018). NAP accelerates leaf and flower senescence through distinct signalling pathways. It is involved in ABA signalling regulation during leaf senescence, and utilizes SA‐ and ROS‐mediated signalling pathways during flower senescence (Fan *et al*., 2015; Meng *et al*., 2022b). It collaborates with CBFs to increase plant susceptibility to cold stress, and it exhibits positive and negative regulation of drought resistance in tomato and *Arabidopsis*, respectively, through the ABA pathway (An *et al*., 2018; Sakuraba *et al*., 2015; Wang *et al*., 2020c). ATAF1 promotes leaf senescence and negatively regulates plant resistance to *Bgh* through the ABA pathway (Jensen *et al*., 2008). Interestingly, ATAF1 regulates salt tolerance in *Arabidopsis* and soybean in opposing roles (Wu *et al*., 2009; Yang *et al*., 2019). ORE1 accelerates leaf senescence under various conditions, such as natural conditions, flooding, salt stress and disease stress, mainly through ethylene signalling (Balazadeh *et al*., 2010; Garapati *et al*., 2015; Yeung *et al*., 2018). In contrast, JUB1 plays an opposite role to other NACs by delaying leaf senescence through the ROS pathway (Zhang *et al*., 2022b). It also promotes late flowering via the GA/BR pathway (Shahnejat‐Bushehri *et al*., 2016), and improves plant tolerance to drought and salt stress (Alshareef *et al*., 2019; Thirumalaikumar *et al*., 2018). Among the five core TFs (OsNAC2, NAP, ATAF1, ORE1 and JUB1), OsNAC2 and ORE1 belong to the NAM subgroup, whereas NAP, ATAF1 and JUB1 belong to the NAP, ATAF and ONAC022 subgroups, respectively. This suggests that members of these four subgroups may serve as crucial factors in balancing growth, development and stress responses.

## Concluding remarks and future directions

The NAC TF family is one of the largest plant‐specific TF families, and its members are continuously being supplemented by transcriptome and genome analyses. NACs are widely involved in plant growth and development, as well as in biotic and abiotic stress regulation. Despite the attention they have received since their discovery in 1996 (Souer *et al*., 1996), a comprehensive review of NAC was only conducted a decade ago (Puranik *et al*., 2012). However, in the last decade, significant progress has been made in identifying and studying NAC proteins associated with growth, development, and stress responses. Accumulating evidence indicates NAC TFs play important biological roles in various plant processes, including development and stress response (Figure 2; Table 1). Unfortunately, apart from their roles in regulating SCW formation and flowering development, the function of NAC TFs in regulating other growth and developmental processes does not seem to be strongly associated with their grouping (Figure 2). Therefore, certain functions of NACs in growth and development cannot be attributed to the specific structural features of any particular subgroup. Nonetheless, the NAP, AtNAC3 and ATAF subgroups widely participate in various stresses, demonstrating that these three subgroups are closely related to stress (Table 1), Further research on the functions of other subgroups is critical. Notably, certain NAC TFs, such as OsNAC2, NAP, ATAF1, ORE1 and JUB1, have been extensively studied as central hubs in plant development and stress response (Figure 4), showcasing their versatile functions in enabling plant survival under diverse stress conditions.

While significant progress has been made, many problems remain unsolved. Firstly, the NAC regulatory network involved in plant development and stress response is not static but should be highly dynamic and complex, in NAC regulatory network, whether there is a hierarchical relationship between NAC TFs and other TFs. If so, who is the upper regulator is also a challenge worth investigating. Secondly, how do NACs sense developmental and stress signals? What mechanisms underlie their involvement in balancing growth and stress responses? How do plants utilize NACs to initiate various processes? Thirdly, what causes functional differentiation within the same NAC subgroup and between different species? What are the reasons why the same NAC gene or some redundant NAC genes in different species play opposite roles in regulation? Furthermore, the innovation of research methods will be very significant to promote the deeper research of NAC TFs. To gain a deeper understanding of the regulation of the NAC network in plant growth and stress responses, combining bioinformatics and systems analyses is crucial., which is of great value in predicting the role of NAC proteins in the regulatory network and inferring their functions in other species. Finally, applying and promoting existing NAC TF research findings in plant germplasm innovation and breeding is highly relevant and valuable.

## Declaration of interests

The authors declare no conflicts of interest.

## Supporting information


**Figure S1** Phylogenetic tree of NAC domains in *Oryza sativa* and *Arabidopsis thaliana*. The unrooted phylogenetic tree of NAC domains was depicted by the CLUSTAL X program and was constructed by the neighbour‐joining method. The numbers beside the branches represent bootstrap values (≥500) based on 1000 replications. The NAC domains were classified into two large groups: Groups I and II. Group I was divided into 14 subgroups (TERN, ONAC022, SENU5, NAP, AtNAC3, ATAF, OsNAC3, NAC2, ANAC011, TIP, OsNAC8, OsNAC7, NAC1 and NAM). Group II was divided into ANAC001, ONAC003, ONAC001, and ANAC063. The Figure data was cited in ‘Comprehensive analysis of NAC family genes in *Oryza sativa* and *Arabidopsis thaliana*’ (Ooka *et al*., 2003).

**Table S1** NAC proteins in *Oryza sativa* and *Arabidopsis thaliana*. The Table data cited from ‘Comprehensive analysis of NAC family genes in *Oryza sativa* and *Arabidopsis thaliana*’ (Ooka *et al*., 2003).
