# Supplementary figures and images for: NACs, generalist in plant life
Source: Plant Biotechnol J. 2023 Aug 25;21(12):2433–57. doi: 10.1111/pbi.14161 (PMC10651149; doi:10.1111/pbi.14161)

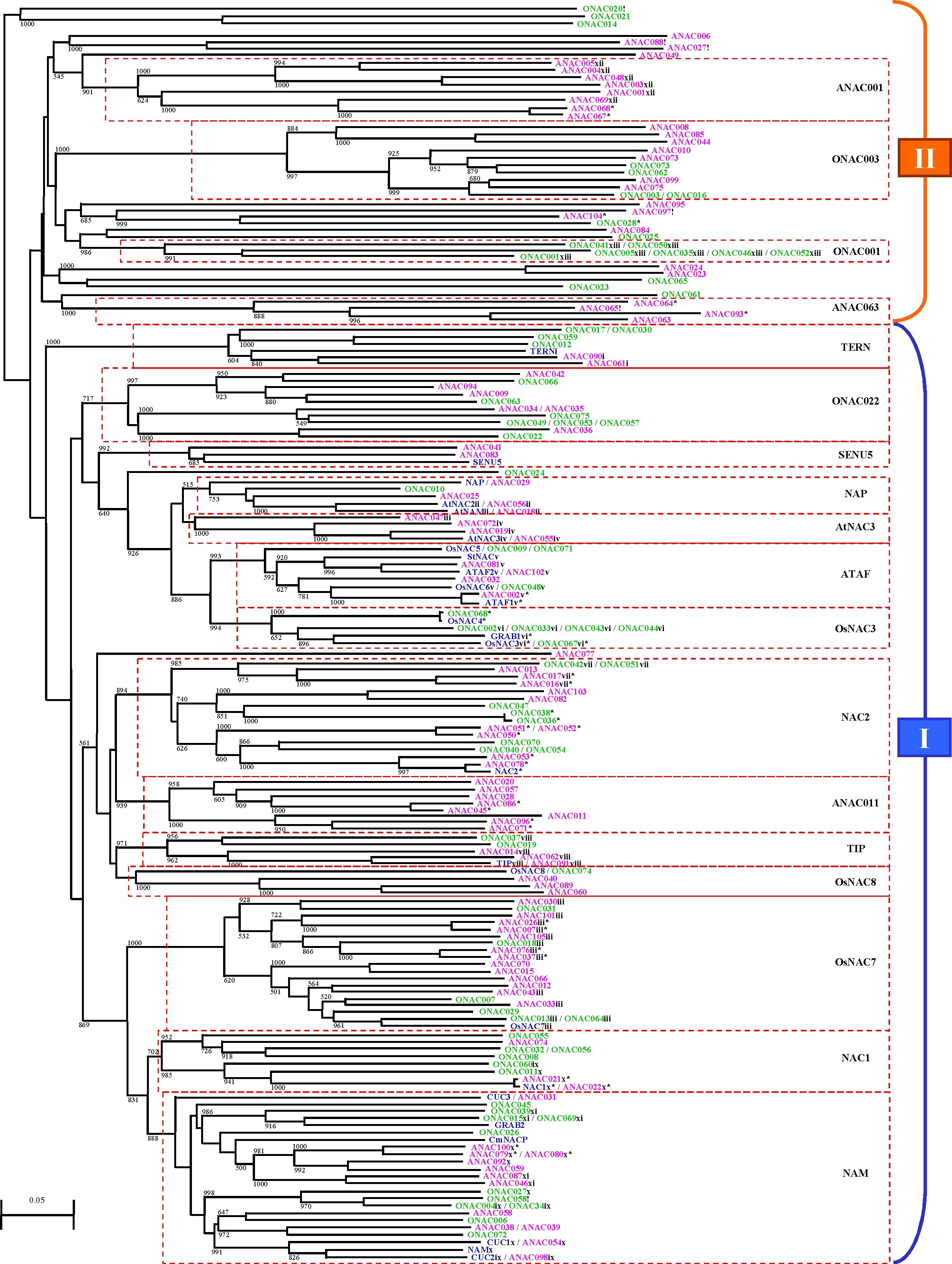

Supplement: Supplementary file 1 — Figure S1 Phylogenetic tree of NAC domains in Oryza sativa and Arabidopsis thaliana. The unrooted phylogenetic tree of NAC domains was depicted by the CLUSTAL X program and was constructed by the neighbour‐joining method. The numbers beside the branches represent bootstrap values (≥500) based on 1000 replications. The NAC domains were classified into two large groups: Groups I and II. Group I was divided into 14 subgroups (TERN, ONAC022, SENU5, NAP, AtNAC3, ATAF, OsNAC3, NAC2, ANAC011, TIP, OsNAC8, OsNAC7, NAC1 and NAM). Group II was divided into ANAC001, ONAC003, ONAC001, and ANAC063. The Figure data was cited in ‘Comprehensive analysis of NAC family genes in Oryza sativa and Arabidopsis thaliana’ (Ooka et al., 2003). [file PBI-21-2433-s002.jpg]
