# Supplementary material for: NACs, generalist in plant life
Source: Plant Biotechnol J. 2023 Aug 25;21(12):2433–57. doi: 10.1111/pbi.14161 (PMC10651149; doi:10.1111/pbi.14161)
Supplement: Supplementary file 2 — Table S1 NAC proteins in Oryza sativa and Arabidopsis thaliana. The Table data cited from ‘Comprehensive analysis of NAC family genes in Oryza sativa and Arabidopsis thaliana’ (Ooka et al., 2003). [file PBI-21-2433-s001.pdf]

Table S1. NAC proteins in *Oryza sativa* and *Arabidopsis thaliana* and other species.

Oryza sativa

Name	Acc. No.	Cluster_ID	Locus (BAC)	Name	Acc. No.	Cluster_ID	Locus (BAC)
ONAC001	AK060509	8702	AC138007	ONAC039	AK065989	1315	AC126222
ONAC002	AK104712	2982	AC135594	ONAC040	AK099629	4933	AP005544
ONAC003	AK061716	1557	AP002743	ONAC041	AK067450	2753	AP004332
ONAC004	AK061745	6131	AL606460	ONAC042	AK099237	3762	AC099403
ONAC005	AK104766	3181	AP005439	ONAC043	AK099245	2982	AC135594
ONAC006	AK062675	9968	AC092780	ONAC044	AK067690	2982	AC135594
ONAC007	AK062952	10226	AP002542	ONAC045	AK067922	3197	AC123525
ONAC008	AK062955	10229	AL606659	ONAC046	AK067906	3181	AP005439
ONAC009	AK063399	12151	AC134047	ONAC047	AK068153	3429	-
ONAC010	AK063406	12158	AP003932	ONAC048	AK068392	3661	AP003561
ONAC011	AK063648	12364	AP004989	ONAC049	AK068393	3662	AP005657
ONAC012	AK063703	12412	AC137611	ONAC050	AK068446	3707	AP004332
ONAC013	AK063943	12596	AP005510	ONAC051	AK068501	3762	AC099403
ONAC014	AK105493	15854	AP003374	ONAC052	AK068776	3181	AP005439
ONAC015	AK105645	11843	AP005167	ONAC053	AK072275	3662	AP005657
ONAC016	AK106152	1557	AP002743	ONAC054	AK069733	4933	AP005544
ONAC017	AK106277	16157	AC112209	ONAC055	AK070416	5571	AC113930
ONAC018	AK106313	16184	AC092389	ONAC056	AK070982	6096	AP005303
ONAC019	AK064178	12776	AP005621	ONAC057	AK071052	3662	AP005657
ONAC020	AK064292	12872	AC134047	ONAC058	AK071020	6131	AL606460
ONAC021	AK106741	16479	AP005839	ONAC059	AK071274	6364	AP003611
ONAC022	AK107090	16730	AC140005	ONAC060	AK071464	6535	AL928780
ONAC023	AK107283	16889	AP004039	ONAC061	AK072682	10946	AC093093
ONAC024	AK107330	16928	AC137617	ONAC062	AK100983	14089	AC124143
ONAC025	AK107369	16958	AC136150	ONAC063	AK073013	11181	AP004700
ONAC026	AK107407	16990	AP006234	ONAC064	AK101280	12596	AP005510
ONAC027	AK108080	17571	AP004766	ONAC065	AK101301	14256	AP005641
ONAC028	AK108454	17913	AP004876	ONAC066	AK073539	11584	AC091494
ONAC029	AK109860	19017	AP004562	ONAC067	AK073667	11686	AP005516
ONAC030	AK109939	16157	AC112209	ONAC068	AK073848	11822	AP004331
ONAC031	AK110611	19701	AP004654	ONAC069	AK073876	11843	AP005167
ONAC032	AK060976	6096	AP005303	ONAC070	AK102173	9590	AP004878
ONAC033	AK104551	2982	AC135594	ONAC071	AK102475	12151	AC134047
ONAC034	AK104626	6131	AL606460	ONAC072	AK102511	14880	AC108757
ONAC035	AK061543	3181	AP005439	ONAC073	AK102794	8554	AP003706
ONAC036	AK065065	356	AC135419	ONAC074	AK102808	9591	AP002817
ONAC037	AK065294	604	AP004698	ONAC075	AK102902	15049	AP003431
ONAC038	AK099540	356	AC135419	Total 56 non-redundant clones			

Arabidopsis thaliana

Name	AGI_code	Name	AGI_code	Name	AGI_code
ANAC001	At1g01010.1	ANAC036	At2g17040.1	ANAC071	At4g17980.1
ANAC002	At1g01720.1	ANAC037	At2g18060.1	ANAC072	At4g27410.2
ANAC003	At1g02220.1	ANAC038	At2g24430.1	ANAC073	At4g28500.1
ANAC004	At1g02230.1	ANAC039	At2g24430.2	ANAC074	At4g28530.1
ANAC005	At1g02250.1	ANAC040	At2g27300.1	ANAC075	At4g29230.1
ANAC006	At1g03490.1	ANAC041	At2g33480.1	ANAC076	At4g36160.1
ANAC007	At1g12260.1	ANAC042	At2g43000.1	ANAC077	At5g04400.1
ANAC008	At1g25580.1	ANAC043	At2g46770.1	ANAC078	At5g04410.1
ANAC009	At1g26870.1	ANAC044	At3g01600.1	ANAC079	At5g07680.1
ANAC010	At1g28470.1	ANAC045	At3g03200.1	ANAC080	At5g07680.2
ANAC011	At1g32510.1	ANAC046	At3g04060.1	ANAC081	At5g08790.1
ANAC012	At1g32770.1	ANAC047	At3g04070.1	ANAC082	At5g09330.1
ANAC013	At1g32870.1	ANAC048	At3g04420.1	ANAC083	At5g13180.1
ANAC014	At1g33060.1	ANAC049	At3g04430.1	ANAC084	At5g14000.1
ANAC015	At1g33280.1	ANAC050	At3g10480.1	ANAC085	At5g14490.1
ANAC016	At1g34180.1	ANAC051	At3g10490.1	ANAC086	At5g17260.1
ANAC017	At1g34190.1	ANAC052	At3g10490.2	ANAC087	At5g18270.1
ANAC018	At1g52880.1	ANAC053	At3g10500.1	ANAC088	At5g18300.1
ANAC019	At1g52890.1	ANAC054	At3g15170.1	ANAC089	At5g22290.1
ANAC020	At1g54330.1	ANAC055	At3g15500.1	ANAC090	At5g22380.1
ANAC021	At1g56010.1	ANAC056	At3g15510.1	ANAC091	At5g24590.2
ANAC022	At1g56010.2	ANAC057	At3g17730.1	ANAC092	At5g39610.1
ANAC023	At1g60280.1	ANAC058	At3g18400.1	ANAC093	At5g39690.1
ANAC024	At1g60350.1	ANAC059	At3g29035.1	ANAC094	At5g39820.1
ANAC025	At1g61110.1	ANAC060	At3g44290.1	ANAC095	At5g41090.1
ANAC026	At1g62700.1	ANAC061	At3g44350.1	ANAC096	At5g46590.1
ANAC027	At1g64105.1	ANAC062	At3g49530.1	ANAC097	At5g50820.1
ANAC028	At1g65910.1	ANAC063	At3g55210.1	ANAC098	At5g53950.1
ANAC029	At1g69490.1	ANAC064	At3g56530.1	ANAC099	At5g56620.1
ANAC030	At1g71930.1	ANAC065	At3g56560.1	ANAC100	At5g61430.1
ANAC031	At1g76420.1	ANAC066	At3g61910.1	ANAC101	At5g62380.1
ANAC032	At1g77450.1	ANAC067	At4g01520.1	ANAC102	At5g63790.1
ANAC033	At1g79580.1	ANAC068	At4g01540.1	ANAC103	At5g64060.1
ANAC034	At2g02450.1	ANAC069	At4g01550.1	ANAC104	At5g64530.1
ANAC035	At2g02450.2	ANAC070	At4g10350.1	ANAC105	At5g66300.1

Other species

Species	Name	Acc. No.	Species	Name	Acc. No.
Petunia hybrida	NAM	X92204	*Triticum sp*	GRAB2	AJ010830
Lycopersicon esculentum	SENU5	Z75524	*Solanum tuberosum*	StNAC	AJ401151
Nicotiana tabacum	TERN	AB021178	*Cucurbita maxima*	CmNACP	Unregistered
Triticum sp	GRAB1	AJ010829			

Predicted NAC proteins in *Oryza sativa* and *Arabidopsis thaliana* were named "ONAC" and "ANAC," respectively. Cluster_ID: from KOME [http://cdna01.dna.affrc.go.jp/cDNA/];[6] Acc. No. and Locus (BAC): from GenBank [http://www.ncbi.nlm.nih. gov/];[20] AGI code: from TAIR [http://www.arabidopsis.org/].[19] ONAC047 does not have a "Locus (BAC)," because it was not mapped.
